# Integrating Molecular Simulations with Machine Learning Guides in the Design and Synthesis of [BMIM][BF_4_]/MOF Composites for CO_2_/N_2_ Separation

**DOI:** 10.1021/acsami.3c02130

**Published:** 2023-03-27

**Authors:** Hilal Daglar, Hasan Can Gulbalkan, Nitasha Habib, Ozce Durak, Alper Uzun, Seda Keskin

**Affiliations:** ‡ Department of Chemical and Biological Engineering, Koç University, Rumelifeneri Yolu, Sariyer, 34450 Istanbul, Turkey; § Koç University TÜPRAŞ Energy Center (KUTEM), Koç University, Rumelifeneri Yolu, 34450 Sariyer, Istanbul, Turkey; ⊥ Koç University Surface Science and Technology Center (KUYTAM), Koç University, Rumelifeneri Yolu, 34450 Sariyer, Istanbul, Turkey

**Keywords:** metal−organic framework, ionic liquid, IL/MOF composite, machine learning, flue gas separation

## Abstract

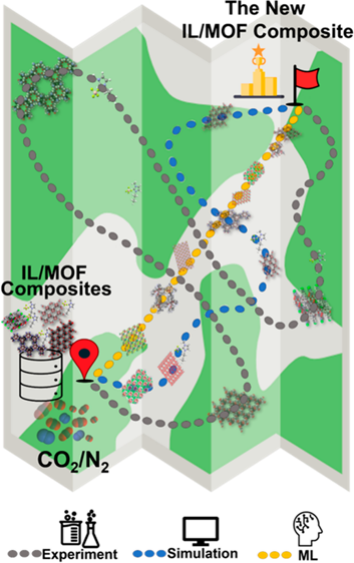

Considering the existence of a large number and variety of metal–organic frameworks (MOFs) and ionic liquids (ILs), assessing the gas separation potential of all possible IL/MOF composites by purely experimental methods is not practical. In this work, we combined molecular simulations and machine learning (ML) algorithms to computationally design an IL/MOF composite. Molecular simulations were first performed to screen approximately 1000 different composites of 1-*n*-butyl-3-methylimidazolium tetrafluoroborate ([BMIM][BF_4_]) with a large variety of MOFs for CO_2_ and N_2_ adsorption. The results of simulations were used to develop ML models that can accurately predict the adsorption and separation performances of [BMIM][BF_4_]/MOF composites. The most important features that affect the CO_2_/N_2_ selectivity of composites were extracted from ML and utilized to computationally generate an IL/MOF composite, [BMIM][BF_4_]/UiO-66, which was not present in the original material data set. This composite was finally synthesized, characterized, and tested for CO_2_/N_2_ separation. Experimentally measured CO_2_/N_2_ selectivity of the [BMIM][BF_4_]/UiO-66 composite matched well with the selectivity predicted by the ML model, and it was found to be comparable, if not higher than that of all previously synthesized [BMIM][BF_4_]/MOF composites reported in the literature. Our proposed approach of combining molecular simulations with ML models will be highly useful to accurately predict the CO_2_/N_2_ separation performances of any [BMIM][BF_4_]/MOF composite within seconds compared to the extensive time and effort requirements of purely experimental methods.

## 1 Introduction

Metal–organic frameworks (MOFs), consisting of metal nodes and organic linkers, have been widely studied for gas separation thanks to their tunable pore sizes and chemical functionalities.^1−3^ One of the major advancements in this field is the incorporation of ionic liquids (ILs) into MOFs via post-synthesis modification techniques^4,5^ to improve the selectivity of MOFs by tuning the relative affinity of composites for a specific molecule, such as CO_2_. Experimental studies have examined a very limited number of IL/MOF composites to date for CO_2_/N_2_,^6−22^ CO_2_/CH_4_,^6−12,14−19,22−24^ and CH_4_/N_2_^6−8,14,17,19^ separations and revealed their high gas separation potentials. There may be many other IL/MOF composites that can outperform the existing porous materials in terms of gas separation performance. However, experimental synthesis, characterization, and testing of all possible IL/MOF composites are impossible considering the very large number of potentially synthesizable ILs (10^18^) and already synthesized MOFs (116,981), yielding more than 10^23^ different composite possibilities.^25,26^

Molecular simulations have been proven to be useful for screening a large number of MOFs,^27,28^ covalent–organic frameworks,^29,30^ and computer-generated hypothetical MOFs (hMOFs)^31,32^ to identify the most selective materials for a target application. However, a very limited number of studies focused on the molecular simulation of IL/MOF composites for gas separations. For instance, using configurational bias Monte Carlo simulations, two different ILs, 1,3-dimethylimidazolium tetrafluoroborate ([MMIM][BF_4_]) and 1-butyl-3-methylimidazolium bis(trifluoromethylsulfonyl)amide ([BMIM][Tf_2_N]), were incorporated into hMOFs, and the most promising materials for CO_2_/CH_4_ separation were identified to have a specific type of topology and pore size.^33^ Grand canonical Monte Carlo (GCMC) simulations were used to study 1085 different types of IL/MOF composites composed of 1-*n*-butyl-3-methylimidazolium tetrafluoroborate ([BMIM][BF_4_]), and many composites exhibiting approximately up to 25 times higher CO_2_/N_2_ selectivity than pristine MOFs were identified.^34^ Exemplified by these results, molecular simulations can be very useful to study a large number and variety of IL/MOF composites and to direct experimental efforts toward the most promising materials. However, simulating gas adsorption in the pores of IL/MOF composites is computationally demanding due to the time and computer-power requirement of IL optimization via density functional theory (DFT) calculations and incorporation of the optimized IL geometry into the pores via equilibrium simulations. Performing molecular simulations for a limited number of representative IL/MOF composites and extracting useful structure–performance relationships from these data using machine learning (ML) algorithms can be a very powerful approach to identify the most important structural properties of the composites, leading to a high gas separation performance. Integrating ML into molecular simulations for developing models that can accurately predict the performances of all possible IL/MOF composites for a target separation in a very short time would be very useful to direct the experimental resources, efforts, and time toward the most promising IL/MOF composites.

In this work, we combined molecular simulations with ML to screen a large number and variety of IL/MOF composites for flue gas separation, which is an industrially, economically, and socially crucial separation to combat global warming. We first focused on a subset of the Cambridge Structural Database (CSD) composed of 941 experimentally synthesized MOFs and computationally incorporated a commercial and low-cost IL, [BMIM][BF_4_], into the pores of each MOF. GCMC simulations were then performed to generate single-component CO_2_ and N_2_ adsorption properties of IL/MOF composites. Simulated gas adsorption data were used to develop the ML models that can accurately predict CO_2_ and N_2_ uptakes and the corresponding CO_2_/N_2_ selectivities based on the structural and chemical properties of the IL/MOF composites. The transferability of the ML models to unseen IL/MOF composites, which were not included in our original data set for training ML models, was examined by comparing the ML-predicted and experimentally measured gas uptakes and selectivities of previously synthesized IL/MOF composites. The most important structural features driving CO_2_/N_2_ selectivity were identified from the ML analysis and used to computationally generate a new composite, [BMIM][BF_4_]/UiO-66, which was not present in our original IL/MOF composite database. This composite was then synthesized, characterized in detail, and tested for adsorption-based CO_2_/N_2_ separation. The synthesis and in-depth characterization of this new [BMIM][BF_4_]/UiO-66 composite and the consequent gas adsorption measurements, including the reproducibility checks, took several months. Setting up and performing molecular simulations to compute the CO_2_ and N_2_ adsorption isotherms of the [BMIM][BF_4_]/UiO-66 composite required several weeks via a high-performance computer cluster. In contrast to these extensive time and effort requirements of experimental work and molecular simulations, predicting CO_2_ and N_2_ adsorption and the separation performance of the [BMIM][BF_4_]/UiO-66 composite by using the ML models that we developed in this work requires only seconds using a personal computer. Therefore, our work demonstrates the strong potential of combining molecular simulations with ML algorithms toward the rational design and development of new IL/MOF composites for various gas separations and has the potential to be extended to other IL-incorporated porous materials and applications.

## 2 Methodology

**Figure 1:**
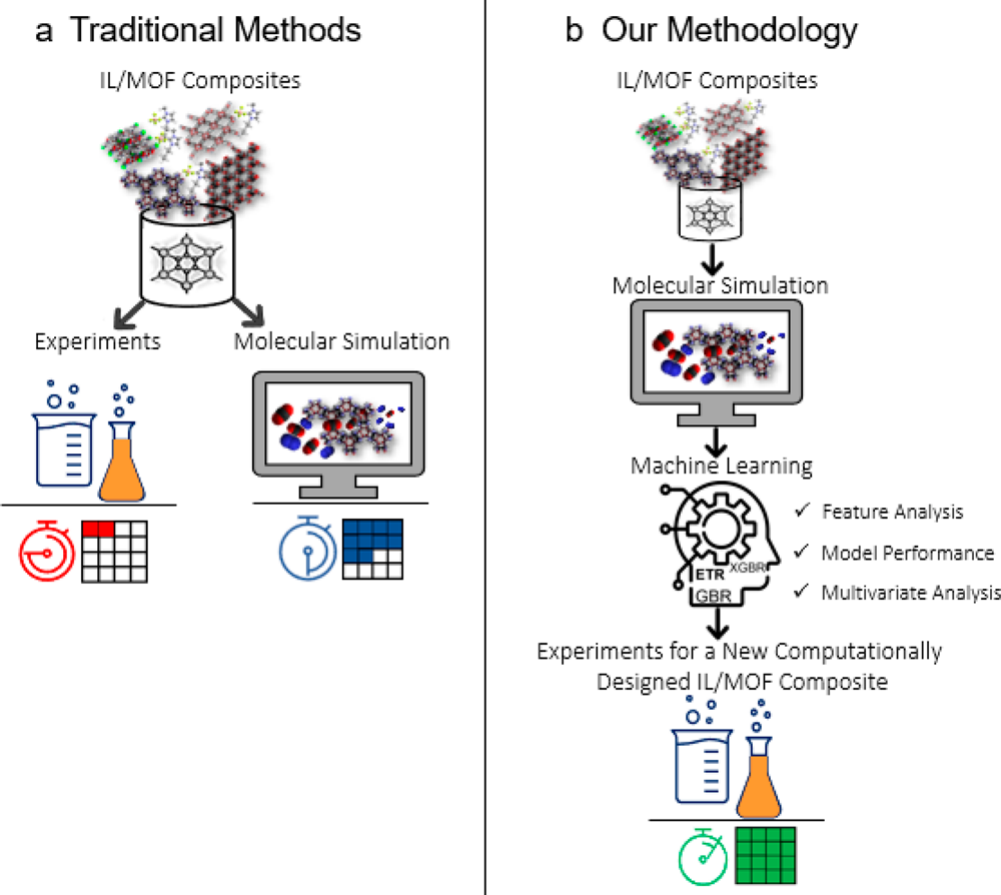
Data-driven design of a new IL/MOF composite. (a) Experimental testing or molecular simulations for all possible IL–MOF combinations. Chronometers roughly represent the time required for assessing the gas separation performance of a single IL/MOF composite. The box represents all possible IL–MOF combinations, and the colored region of the boxes represents the number of IL/MOF composites that can be studied using corresponding methods. (b) Methodology that we proposed in this work combining molecular simulations, ML, and experiments. Molecular simulations were performed for many IL/MOF composites to obtain gas adsorption data, and simulation results were used to develop ML models that can accurately predict the gas separation performance of IL/MOF composites in a time-efficient manner. ML predictions were then used to computationally design a new IL/MOF composite, which was then experimentally synthesized, characterized, and tested for a target gas separation.

To date, only 49 different types of IL/MOF composites composed of 35 different ILs and 6 different MOFs have been experimentally studied for CO_2_ separations, as listed in Table S1. Studying all possible IL–MOF combinations for a target gas separation using purely experimental techniques or using solely molecular simulations, as shown in Figure 1a, is not possible. Our methodology combining molecular simulations and ML for the computational design of a new IL/MOF composite followed by experiments is presented in Figure 1b. We aimed to study many different types of MOFs with one type of IL to construct a wide variety of IL/MOF composite data sets. Therefore, we focused on the CSD nondisordered MOF database^35^ and filtered materials to identify the MOFs having a pore limiting diameter (PLD) of > 6 Å and an accessible surface area (ASA) of > 0 m^2^/g to ensure that the IL molecules can be successfully incorporated into the MOF.^8^ As a result of this filtration, we ended up with 941 different types of MOFs.

Geometry optimization on [BMIM][BF_4_], a well-studied IL, was performed by using the *Gaussian16* program package.^36^ For this purpose, first, a conformer search was performed on different anion–cation pair configurations to determine the energetically most stable ion-pair configuration by DFT calculations. All possible conformations were located using the Becke-three-parameter–Lee–Yang–Parr (B3LYP) functional, including Grimme’s D2 correction and 6-31+G(d) basis set.^37,38^ Next, Baker’s minimization approach^39^ with the *NVT* ensemble was used to incorporate the optimized [BMIM][BF_4_] geometry into the pores of each MOF, as implemented in the *RASPA*(40) software, version 2.0.37. In the minimization procedure, the atomic coordinates and partial charges of the IL atoms were obtained from DFT calculations. Full natural bond orbital (NBO) analysis with *NBO*, version 3.1, was used to calculate the partial charges of the IL using population analyses.^41,42^ The IL loading of the composites was set to one IL molecule per unit cell of a MOF corresponding to 0.15–28.60 wt % IL loading for 941 different types of composites.

Single-component CO_2_ and N_2_ adsorptions in MOFs and IL/MOF composites were computed by performing GCMC simulations at 1 bar and 298 K. Ideal CO_2_/N_2_ selectivities of the materials were calculated as the ratio of the adsorbed amount of CO_2_ to that of N_2_. To quantify the affinity of MOFs and IL/MOF composites to the gas molecules, isosteric heat of adsorption (*Q*_st_^0^) values of the gases were computed at infinite dilution at 298 K using the Widom particle insertion method.^43^ All details of GCMC simulations, such as the type of force field, the charge assignment method used for the materials and gases, and the types of moves are given in the Supporting Information (SI).

The structural properties of MOFs and [BMIM][BF_4_]/MOF composites, such as the PLD, the largest cavity diameter (LCD), ASA, geometric pore volume (PV), porosity (ϕ), and density (ρ) were computed using *Zeo++* software.^44^ Chemical descriptors, such as the total degree of unsaturation (TDU), carbon percentage (C%), and hydrogen percentage (H%), were calculated from the crystal information file (CIF) of the structures. A total of 19 and 20 different easily achievable features were used for MOFs and IL/MOF composites, respectively. Textural properties (PLD and LCD), chemical properties (degree of unsaturation (DU), TDU, oxygen-to-metal ratio (O-to-M)), energy-based descriptors (*Q*_st_^0^), and IL% for IL/MOF composites were used as the inputs of ML models (Table S2).

Simulated CO_2_ and N_2_ adsorption properties of MOFs and IL/MOF composites were used as the outputs to develop ML models with a training set of 80% and a test set of 20% for MOF and IL/MOF data sets, respectively. As a result, four different ML models were developed to predict CO_2_ and N_2_ uptakes of MOFs and IL/MOF composites at 1 bar and 298 K using the tree-based pipeline optimization tool (TPOT).^45^ For the development of the ML models, the Random Forest,^46,47^ the Extra Tree,^48^ the GradientBoosting,^49^ and the Extreme Gradient Boosting (XGB)^50^ regressor algorithms were used with their optimized hyperparameters, as listed in Table S3. ML-predicted gas adsorption data of MOFs and IL/MOF composites were compared with the GCMC simulation results using the coefficient of determination (*R*^2^), mean absolute error (MAE), root-mean-square error (RMSE), and the Spearman rank-order correlation coefficient (SRCC) to assess the accuracy of the models. To investigate the predictability power of ML models for the gas separation performance of the materials, we compared the ML-predicted CO_2_/N_2_ selectivities of the materials with the GCMC-simulated ones. The transferability of the ML models was tested by predicting CO_2_ and N_2_ uptakes of the previously synthesized [BMIM][BF_4_]/MOF composites reported in the literature: [BMIM][BF_4_]/ZIF-8 (7.5 wt %)^34^ and [BMIM][BF_4_]/Cu-BTC (30 wt %),^8^ which were not in the training set of our ML models. Based on this ML analysis, structural, chemical, and energy-based features that can potentially lead to high selectivity were identified and used to computationally generate the new [BMIM][BF_4_]/UiO-66 composite, which was not present in our original IL/MOF composite data set used for training of ML models.

Finally, we synthesized the [BMIM][BF_4_]/UiO-66 (10.4 wt %) composite by using wet-impregnation method^51^ and characterized it in detail using Brunauer–Emmett–Teller (BET) analysis, X-ray diffraction (XRD), scanning electron microscopy (SEM) with energy-dispersive X-ray (EDX) spectroscopy, and infrared (IR) spectroscopy. Volumetric single-component CO_2_ and N_2_ adsorption measurements were performed for pristine UiO-66 and [BMIM][BF_4_]/UiO-66 composite at 1 bar and 298 K, and compared with the results of molecular simulations and ML predictions. All details about performing the data preprocessing and molecular simulations, selectivity calculations, computation of the structural and chemical features of the materials, development of ML models, synthesis, characterization, and gas adsorption measurements of the [BMIM][BF_4_]/UiO-66 composite are provided in the SI. All of the codes and data that we used to develop the ML models are provided on GitHub (https://github.com/hdaglar/BMIM.BF4.MOF_Composites_ML).

## 3 Results and Discussion

### 3.1 Development of ML Models for IL/MOF Composites

**Figure 2:**
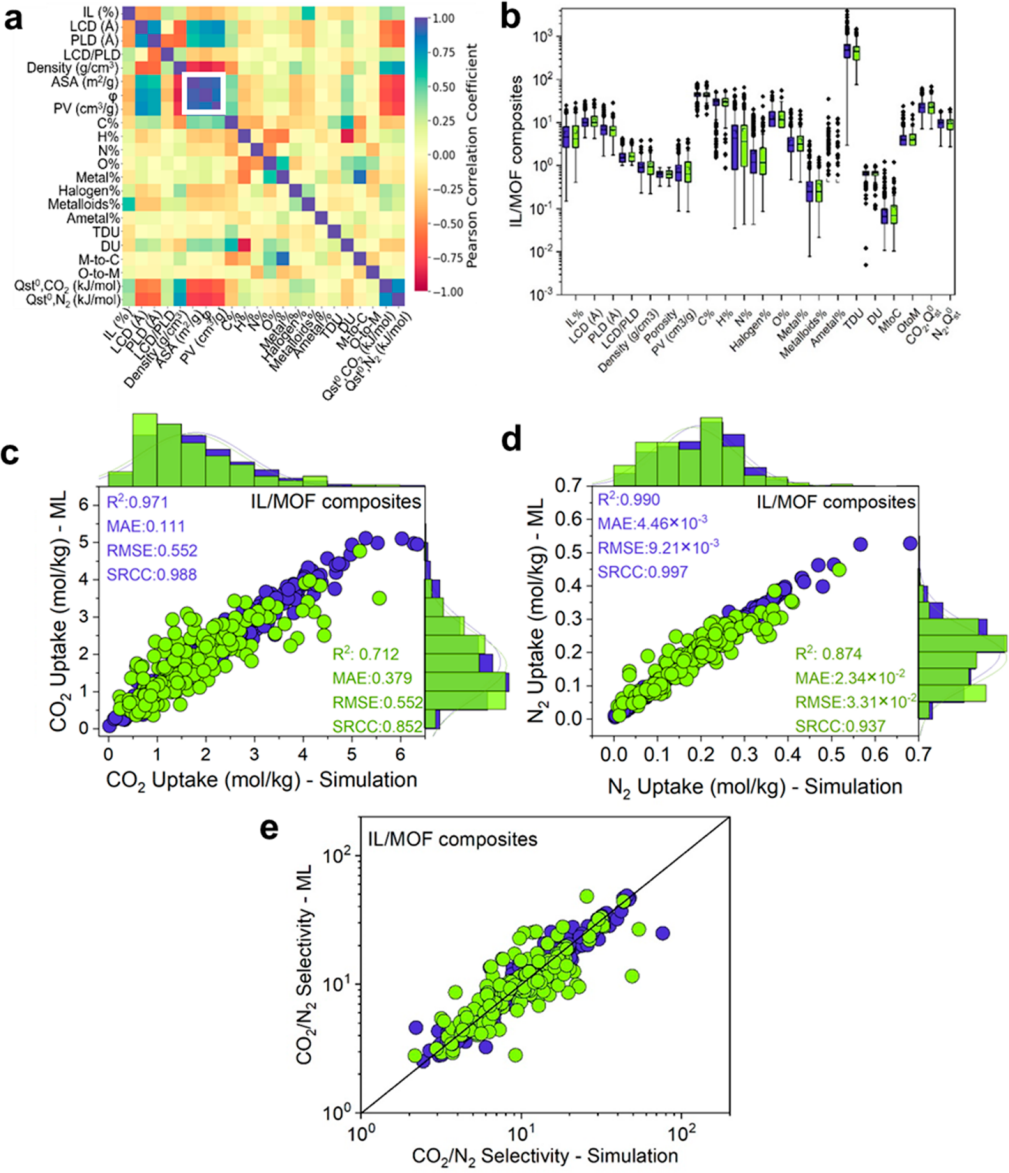
ML models for IL/MOF composites. (a) Correlation heatmap of the features of all IL/MOF composites. The strong correlation between the ASA, PV, and porosity is highlighted with the white box. (b) Box plots showing the distribution of features based on the training and test sets for IL/MOF composites. Comparison of ML predictions with the GCMC results for single-component (c) CO_2_ and (d) N_2_ uptakes of [BMIM][BF_4_]/MOF composites at 1 bar and 298 K. Marginal histograms show the distribution of gas uptake data in the training and test sets. Training and test sets are represented as purple and green symbols, respectively, in parts b–e. (e) Comparison of the ML-predicted and GCMC-simulated selectivities of IL/MOF composites.

We first focused on the structural, chemical, and energy-based features of the [BMIM][BF_4_]/MOF composites and examined the correlations between each feature used as the input of ML models. The heatmaps with Pearson correlations (*r*) across different features of [BMIM][BF_4_]/MOF composites are shown in Figure 2a. Because there is a very strong correlation between the ASA and pore volume (*r* = 0.91) and between the ASA and porosity (*r* = 0.93), we did not use ASA as a descriptor to avoid multicollinearity problems (*r* > 0.90). We then analyzed the univariate relationship between the materials’ features and their gas adsorption properties. Results showed that some features of [BMIM][BF_4_]/MOF composites and MOFs correlate with their corresponding CO_2_ and N_2_ uptakes to some extent (Figures S1 and S2). However, univariate analysis is insufficient to elucidate the relationships between several other features and the gas adsorption properties of the materials, highlighting the need for multivariate analysis to reveal the hidden structure–performance relationships. We used 20 different features (LCD, PLD, LCD/PLD, porosity, PV, density, IL wt %, C%, H%, N%, O%, TDU, DU, O-to-M, M-to-C, halogen%, metalloids%, ametal%, metal%, and *Q*_st_^0^) in training the ML models, as listed in Table S2.

We then showed that the feature distribution in the training and test sets have similar characteristics for 941 different types of IL/MOF composites (Figure 2b). Parts c and d of Figure 2 illustrate the scatter plots with marginal histograms for the relationships between the ML-predicted and GCMC-simulated gas adsorption data of IL/MOF composites. The ML-predicted CO_2_ and N_2_ uptakes of the composites have a good correlation with the GCMC results, supported by the high *R*^2^ values in the range of 0.71–0.87 and low RMSE (3.3 × 10^–2^–0.55) and MAE (2.3 × 10^–2^–0.38) values for the test sets. In Figure 2c,d, the RMSE values are higher than the MAE values because our data set includes some IL/MOF composites that exhibit a higher gas uptake relative to the nearest data point. Since the maximum gas uptake values used to train tree-based ML algorithms also affect the upper limits of ML predictions, we kept these structures in our database. Our work mainly aims to predict the CO_2_/N_2_ separation performances of IL/MOF composites. Therefore, ML predictions for pristine MOFs that we considered in this work are provided in the SI. The correlation map of the feature distribution and models’ accuracy are presented in Figure S3. A total of 19 features that we described above were used as inputs of ML models to predict gas adsorption in MOFs, shown in Figures S3a,b, and the feature distribution in the training and test sets were similar. Parts c and d of Figure S3 show that the calculated *R*^2^ (0.77–0.89), RMSE (3.3 × 10^–2^–0.59), MAE (2.14 × 10^–2^–0.36), and SRCC (0.92–0.94) values of the ML models developed for MOFs were similar to the values calculated for the IL/MOF composites.

We also showed the ratios of the ML-predicted gas uptakes to the simulated ones for the test sets of ML models developed for IL/MOF composites and MOFs in Figure S4. The average ratio is close to unity (in the range of 0.7–1.3 with a discrepancy of ±30%) for each case, indicating the good agreement between ML and simulations. However, ML predictions for some MOFs significantly overestimate or underestimate (discrepancy > ±30%) simulation results: ML predictions for 56 and 26 IL/MOF composites (48 and 21 MOFs) exhibit >±30% discrepancy for CO_2_ and N_2_ uptakes, respectively. In Figure S4, the deviations are more observable for CO_2_ uptake because its adsorption is more complex due to the presence of strong electrostatic interactions between CO_2_ and IL/MOF composites. Parts c and d of Figure 2 also show that ML models generally underestimate the simulation results, especially for composites exhibiting high gas uptakes. This result can be attributed to the fact that only a small number of composites show high gas uptakes (>3.5 mol/kg for CO_2_ and >0.5 mol/kg for N_2_) among the composites used to develop the ML models. It is also important to note that supervised ML algorithms, such as XGB, struggle with extrapolating the data, and these ML algorithms generally make a good prediction for the gas uptake range previously seen in the training data set.^52^ CO_2_ and N_2_ adsorption predictions for the IL/MOF composites exhibit high SRCC ranges in the test set (0.85 and 0.94, respectively), suggesting that the ranking of materials based on the ML-predicted adsorption properties is highly similar to the rankings based on the GCMC-simulated ones.

One of the main goals of this work is to develop accurate ML models for predicting the CO_2_/N_2_ selectivities of [BMIM][BF_4_]/MOF composites in a time-efficient manner. Therefore, we compared the ML-predicted CO_2_/N_2_ selectivities of IL/MOF composites with the GCMC-simulated ones in Figure 2e. We note that the “ML-predicted selectivity” represents the selectivity calculated by using ML-predicted CO_2_ and N_2_ uptake data, and the “GCMC-simulated selectivity” indicates the selectivity computed by using CO_2_ and N_2_ uptake data obtained from GCMC simulations. Figure 2e shows that the ML-predicted selectivities of IL/MOF composites agree well with the GCMC-simulated ones at 1 bar and 298 K. The range of the ML-predicted selectivities (2.2–54.2) is similar to that of the GCMC-simulated ones (2.8–48.2) in the test set, suggesting that ML models are useful to accurately assess the gas separation performances of IL/MOF composites that exist in our database.

**Figure 3:**
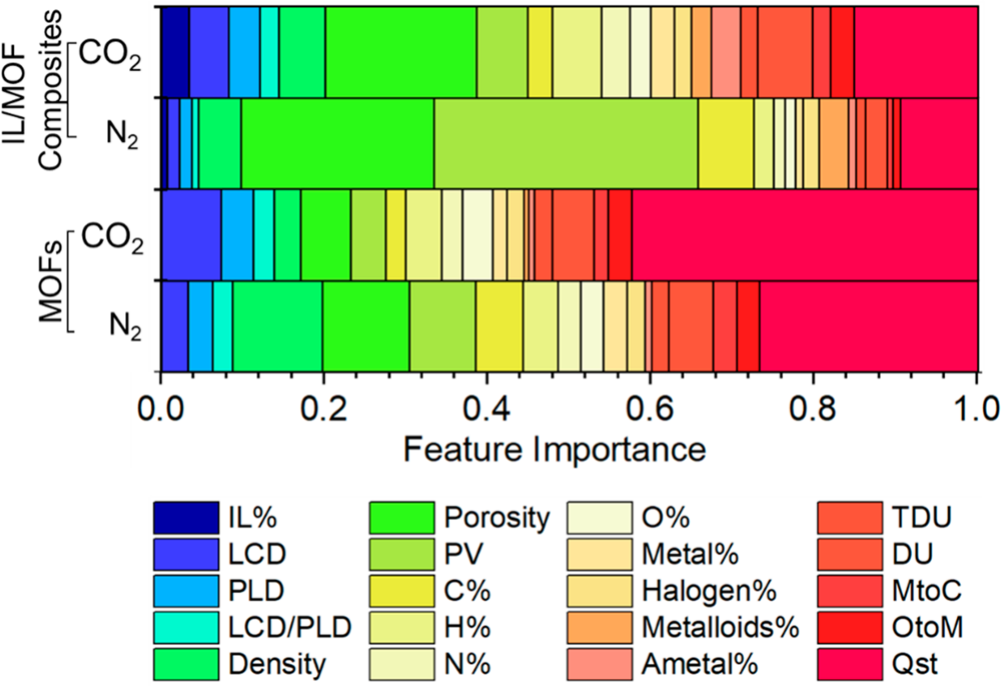
Interpretability of the ML models. Feature importance analysis for the gas adsorption properties of [BMIM][BF_4_]/MOF composites and pristine MOFs. The width range of each color shows the importance of the related feature. The colors are taken from the same palette for each gas adsorption property.

An important benefit of developing a ML model for predicting the materials’ target data is to gain molecular-level insight into the contribution of features to these target properties. The feature importance analysis presented in Figure 3 demonstrates that the most important features of the IL/MOF composites that determine their gas uptakes are *Q*_st_^0^ for CO_2_ adsorption, the pore volume for N_2_ adsorption, and the porosity for both CO_2_ and N_2_ adsorptions. This analysis suggests that the combination of structural features is more pronounced than energy-based features for determining the gas adsorption properties of the IL/MOF composites. The feature importance analysis performed for pristine MOFs showed that *Q*_st_^0^ is the most important descriptor to predict CO_2_ and N_2_ uptakes of MOFs, and this descriptor is more pronounced for CO_2_ than for N_2_. This result is expected because the N_2_ molecule has weaker electrostatic interactions with the framework compared to CO_2_. The most important message that can be obtained by comparing the feature importance analysis of MOFs and IL/MOF composites is that material features related to the pore geometry become more dominant in determining the gas adsorption properties after IL incorporation because the pore sizes of the IL/MOF composites become much smaller than those of pristine MOFs, leading to a more confined environment.

**Figure 4:**
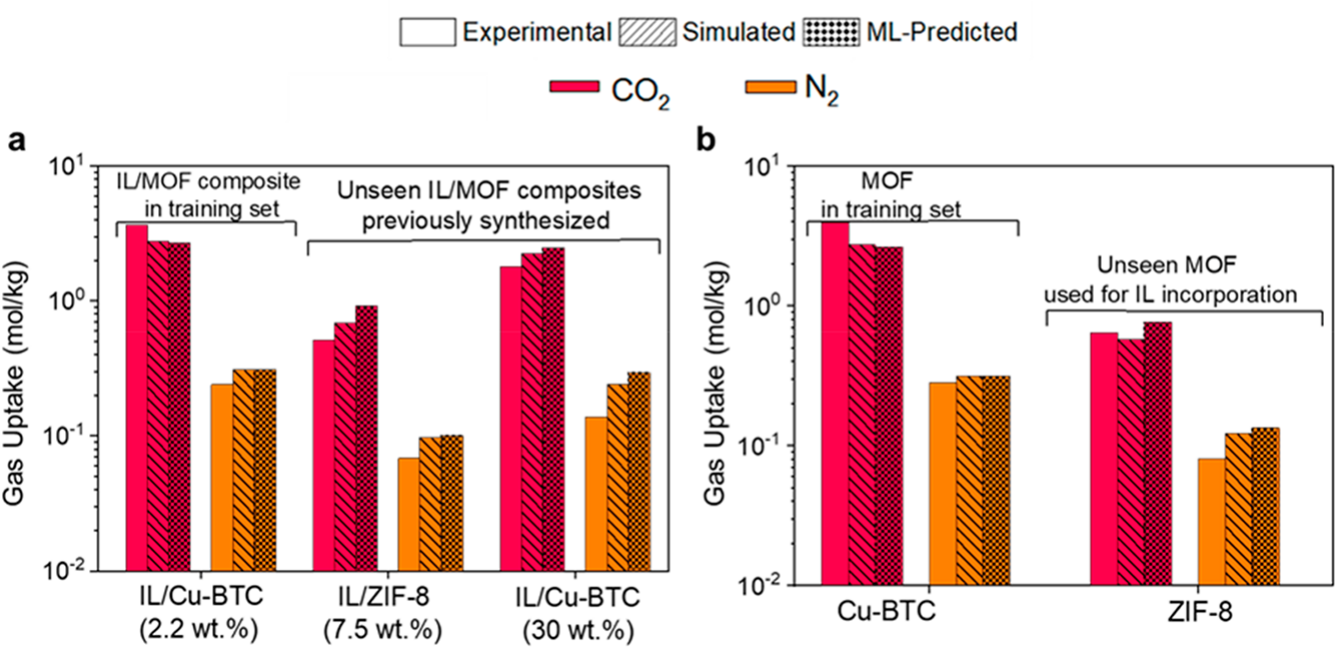
Transferability of the ML models. Comparison of the experimental, simulated, and ML-predicted CO_2_ and N_2_ adsorption data of previously synthesized (a) [BMIM][BF_4_]/Cu-BTC and [BMIM][BF_4_]/ZIF-8 composites and (b) pristine MOFs (Cu-BTC and ZIF-8) at 1 bar and 298 K.

We trained the ML models using the GCMC simulation results of 941 different types of MOFs and their [BMIM][BF_4_] incorporated composites and tested the accuracy of ML models by comparing the ML-predicted gas adsorption properties of the materials with the results of GCMC simulations. To investigate the validity of our ML models for any [BMIM][BF_4_]/MOF composite, including those outside of the database that we used for the ML model development, we collected CO_2_ and N_2_ uptake data of experimentally reported [BMIM][BF_4_]/MOF composites and their corresponding pristine MOFs from the literature. Figure 4a shows experimental, GCMC-simulated, and ML-predicted data of three different [BMIM][BF_4_]/MOF composites with different IL loadings: 2.2 wt % for Cu-BTC,^34^ 7.5 wt % for ZIF-8,^34^ and 30 wt % for Cu-BTC.^8^ ML-predicted gas uptakes for the [BMIM][BF_4_]/Cu-BTC composite (2.2 wt %) strongly agree with the GCMC-simulated and experimentally measured data because this is one of the composites used to train our ML models. [BMIM][BF_4_]/ZIF-8 (7.5 wt %) and [BMIM][BF_4_]/Cu-BTC (30 wt %) were unseen composites, and they were not in the training and/or test sets that we used to develop the models. ML predictions for [BMIM][BF_4_]/ZIF-8 having 7.5 wt % IL loading slightly overestimated the GCMC-simulated and experimentally reported data. Although our ML models were trained using the data of composites having <30 wt % IL loading (the IL loading was calculated to be in the range of 0.15–28.60 wt % as discussed above), predicted gas uptakes of [BMIM][BF_4_]/Cu-BTC with 30 wt % IL loading were in good agreement with the experimental data, as shown in Figure 4a.

Experimentally measured, GCMC-simulated, and ML-predicted CO_2_ and N_2_ uptakes of two pristine MOFs (Cu-BTC and ZIF-8) that we used to generate [BMIM][BF_4_]/MOF composites agreed well, as shown in Figure 4b. Although some features of unseen materials are out of the corresponding feature distribution of our material database [i.e., the PLD (3.3 Å) of ZIF-8 is out of the PLD distribution (6–31 Å) of MOFs used to develop our models], the ML models still accurately predicted the CO_2_ and N_2_ uptakes and the CO_2_/N_2_ selectivities of unseen IL/MOF composites and their corresponding pristine MOFs. Overall, these results confirmed the transferability of the ML models to unseen materials having different features and highlighted that the models that we developed can be used for the accurate estimation of CO_2_ and N_2_ uptakes of various types of IL/MOF composites and MOFs.

### 3.2 ML-Guided Generation of a New IL/MOF Composite

**Figure 5:**
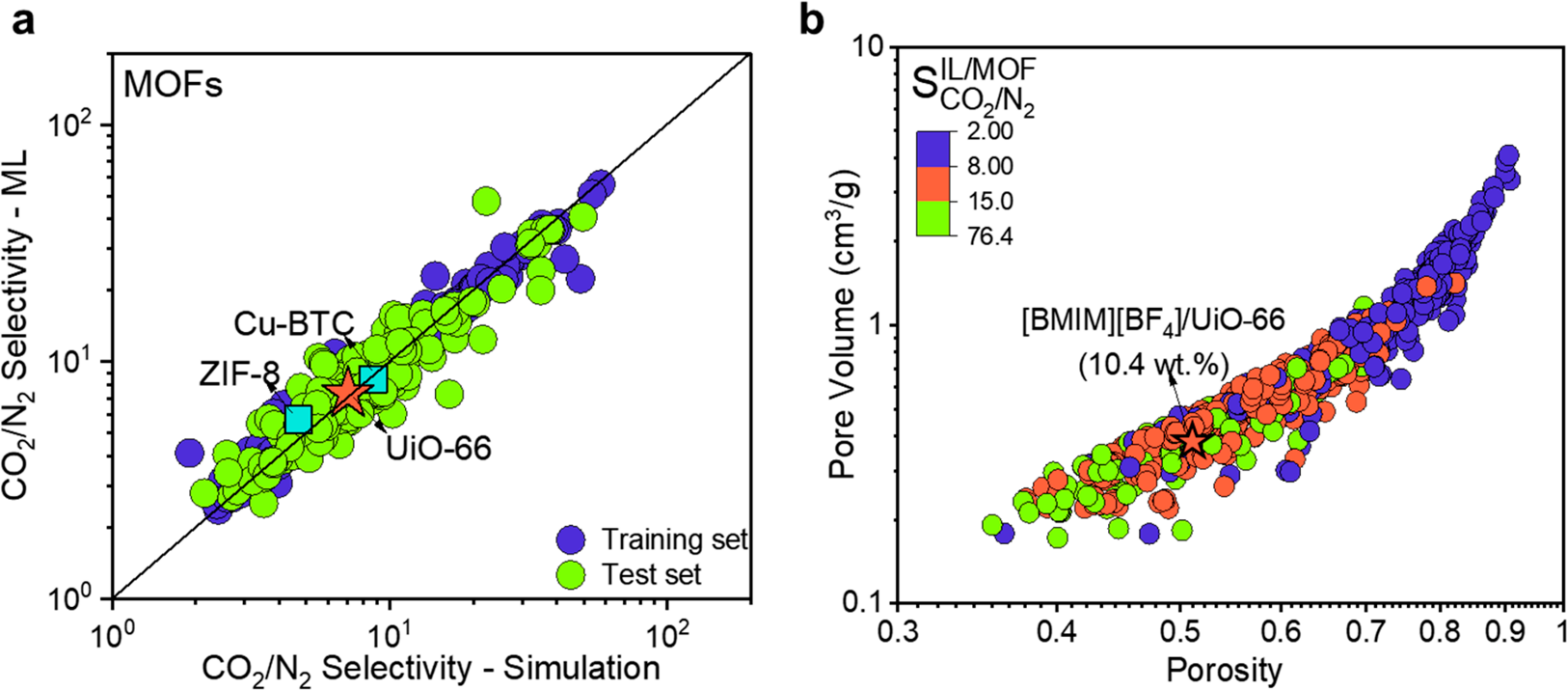
ML-guided generation of the [BMIM][BF_4_]/UiO-66 composite. (a) Comparison of the ML-predicted and GCMC-simulated selectivities of MOFs. Blue boxes represent the CO_2_/N_2_ selectivity of pristine MOFs previously used to synthesize IL/MOF composites. The star represents UiO-66 as a new host for IL incorporation. (b) Relationship between the most important features (pore volume and porosity) and ML-predicted selectivities of 941 different types of [BMIM][BF_4_]/MOF composites. The star represents the pore volume and porosity of a computationally designed [BMIM][BF_4_]/UiO-66 composite having 10.4 wt % IL loading.

The main target of this work is to integrate ML into the molecular simulations of IL/MOF composites to precisely direct experimental efforts to the synthesis of new composites for CO_2_/N_2_ separation. To achieve this, we first focused on the CO_2_/N_2_ selectivity of pristine MOFs to identify a host material for IL incorporation. Figure 5a shows that the ML-predicted selectivities of pristine MOFs strongly agree with the GCMC-simulated ones at 1 bar and 298 K, suggesting that ML models are useful to accurately assess the gas separation performances of pristine MOFs that exist in our database. We compared the ML-predicted selectivities of Cu-BTC and ZIF-8, two hosts that were previously used to make [BMIM][BF_4_]/MOF composites, with the GCMC-based selectivities, and the good agreement confirmed the validity of ML models to accurately estimate the CO_2_/N_2_ selectivity of any type of MOF. To further show the consistency between ML predictions and the results of simulations and experimental measurements, we identified the top three MOFs (refcodes: QOVDEL,^53^ REQBAS,^54^ and FUSMEM^55^) exhibiting the highest CO_2_/N_2_ selectivity in the test set. Table S4 shows CO_2_ and N_2_ uptakes and the corresponding CO_2_/N_2_ selectivities of these MOFs obtained from simulations and ML models. These MOFs exhibit high CO_2_/N_2_ selectivity in the range of 38–50, and they are good candidates for making [BMIM][BF_4_]-incorporated composites. However, to the best of our knowledge, none of these MOFs are commercially available. Hence, we specifically aimed to focus on a commercially available MOF (i) to avoid potential reproducibility problems^56^ in synthesizing these materials and (ii) to prove the transferability of our ML models and the validity of our methodology by studying a MOF that was not present in our original data set yet commercially available at a certain quality through reliable vendors. Thus, we chose a zirconium-based MOF, UiO-66, as the host material for the following practical and theoretical reasons: UiO-66 is robust^57^ and offers high chemical stability and moisture resistance. To confirm the latter, we calculated Henry’s coefficient for water, *K*_H_2_O_ = 2.3 × 10^–6^ mol/kg/Pa for UiO-66, which was lower than the corresponding value of a well-known moisture stable ZIF-8, *K*_H_2_O_ = 5.0 × 10^–6^ mol/kg/Pa.^58^ Therefore, UiO-66 is expected to preserve its stability during wet flue gas separation under real operating conditions. Figure 5a shows that the ML-predicted CO_2_/N_2_ selectivity of pristine UiO-66 strongly agrees with the simulated ones. Theoretical reasons for selecting UiO-66 as the host material of the composite were obtained from the detailed feature importance analysis of ML models given in Figure 3. Pore volume and porosity are the two important features that determine the gas adsorption properties of the IL/MOF composites. Figure 5b shows that a good CO_2_/N_2_ selectivity in the range of 8–15 can be achieved if the [BMIM][BF_4_]/UiO-66 composite with an IL loading of 10.4 wt % is generated, leading to a porosity of 0.35 and a pore volume of 0.51 cm^3^/g. The PLD of this new composite was calculated as 3.66 Å, so that both CO_2_ (3.3 Å) and N_2_ (3.64 Å) molecules can adsorb into the pores of the composite. It is important to note that the new composite has a large enough PLD for the entrance of both gas molecules, independent of their corresponding solubilities in the IL, so that molecular simulations can compute the adsorption of gas molecules, which are modeled as hard spheres.

### 3.3 Synthesis, Characterization, and Adsorption Measurements of [BMIM][BF_4_]/UiO-66

The [BMIM][BF_4_]/UiO-66 composite was synthesized by using the wet-impregnation method, as reported previously^9,19,51^ and then characterized in detail. All details of the synthesis and characterization of the [BMIM][BF_4_]/UiO-66 composite are discussed in the SI. IL loading of the synthesized [BMIM][BF_4_]/UiO-66 composite was estimated by washing the composite several times with acetone. The washed composite was dried overnight at 80 °C, and the IR spectrum of the dried [BMIM][BF_4_]/UiO-66 composite was recorded (Figure S5). A comparison presented in Figure 5 showed that the IR spectrum of the composite was identical to that of pristine UiO-66, confirming the removal of IL molecules. The washed [BMIM][BF_4_]/UiO-66 composite was then weighed, and the IL loading was determined as 9.2 ± 0.6 wt %, which is close to the IL loading of the computationally generated composite (10.4 wt %). The slight difference can be attributed to the loss of some IL on the walls of the sample container during the synthesis process.

**Figure 6:**
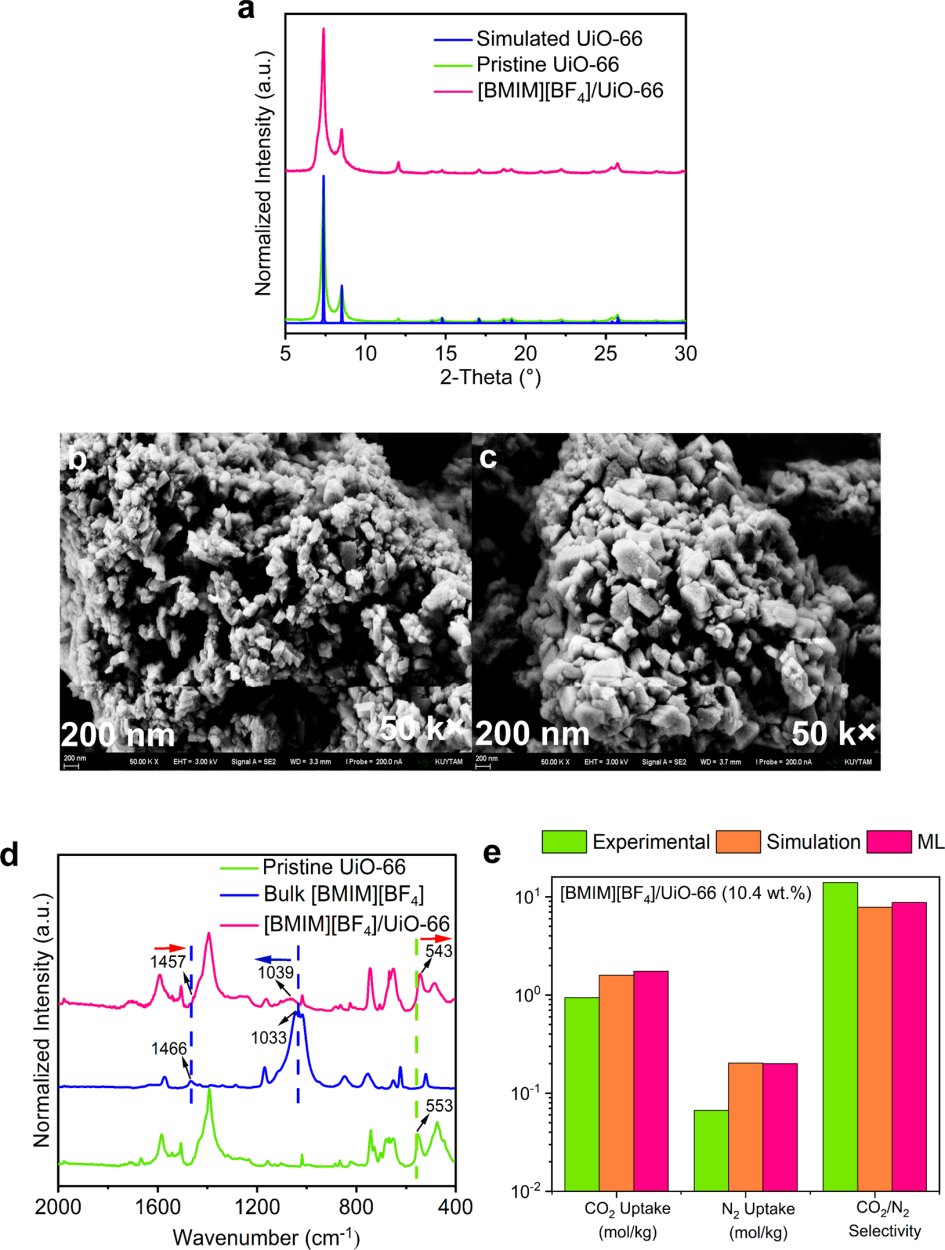
Synthesis, characterization, and testing of the [BMIM][BF_4_]/UiO-66 composite. (a) XRD patterns of simulated and pristine UiO-66 and [BMIM][BF_4_]/UiO-66 in the 2θ range of 5–30°. SEM images of (b) pristine UiO-66 and (c) [BMIM][BF_4_]/UiO-66 composite acquired at a magnification of 50 k×. (d) IR spectra of pristine UiO-66, bulk [BMIM][BF_4_], and the [BMIM][BF_4_]/UiO-66 composite in the spectral range of 2000–400 cm^–1^. (e) Comparison of the experimental, GCMC-simulated, and ML-predicted CO_2_ and N_2_ uptakes and CO_2_/N_2_ selectivities of the [BMIM][BF_4_]/UiO-66 composite at 1 bar and 298 K.

The integrity of the crystalline structure of [BMIM][BF_4_]/UiO-66 composite was investigated by XRD analysis, and no significant change in the XRD data of pristine UiO-66 was observed, as shown in Figure 6a, indicating that the crystalline structure of UiO-66 remained mostly intact upon IL incorporation, consistent with the previous studies.^17,59^ Similarly, no alterations were observed in the SEM images of the [BMIM][BF_4_]/UiO-66 composite obtained at two different magnifications, 50 k× and 10 k×, as presented in Figures 6b,c and S6, respectively, verifying the integrity of the surface morphology upon the incorporation of IL molecules. Moreover, the presence of boron (B) and fluorine (F) elements, inherited from [BMIM][BF_4_] molecules, was verified by the EDX analysis (Figure S7). The nature of the molecular interactions between bulk [BMIM][BF_4_] and UiO-66 was elucidated using IR spectroscopy, as presented in Figure 6d. In summary, the characteristic IR bands of pristine UiO-66 illustrated blue shifts for the vibrational modes of Zr–(OC) and Zr–O, and the cation of the IL, [BMIM]^+^, showed red shifts upon the incorporation of IL into UiO-66.^6^ Hence, these shifts confirm the presence of the intermolecular interactions between the [BMIM]^+^ cation of [BMIM][BF_4_] and the Zr metal nodes of UiO-66. The N_2_ adsorption–desorption isotherm of the [BMIM][BF_4_]/UiO-66 composite (Figure S8) showed a reduction in both the surface area and pore volume (Table S5) compared to that of pristine UiO-66, indicating the successful incorporation of IL molecules into the MOF pores, consistent with the studies of previously reported IL/MOF composites.^6,9,59^ Moreover, conductor-like screening model for realistic solvents (COSMO-RS) solubility calculations (Figure S9) demonstrated that N_2_ has almost negligible solubility in [BMIM][BF_4_] at the BET measurement conditions.

We finally measured the CO_2_ and N_2_ adsorption isotherms by using a volumetric gas sorption analyzer. The CO_2_ adsorption isotherm for pristine UiO-66 was fitted to the dual-site Langmuir model, while the N_2_ isotherm of pristine UiO-66 and the CO_2_ and N_2_ adsorption isotherms of the [BMIM][BF_4_]/UiO-66 composite were fitted to the dual-site Langmuir–Freundlich model. The corresponding fitting parameters are given in Table S6. Because the [BMIM][BF_4_]/UiO-66 composite was selected based on the multivariate analysis obtained from the ML models that predict gas uptakes, we compared the experimentally measured, GCMC-simulated and ML-predicted CO_2_ and N_2_ uptakes and CO_2_/N_2_ selectivities of the [BMIM][BF_4_]/UiO-66 composite at 1 bar and 298 K. Figure 6e shows that while the experimental, simulation, and ML results are in good agreement for CO_2_ uptake of [BMIM][BF_4_]/UiO-66, simulations and ML overestimate the experimentally measured N_2_ uptake, leading to a slight underestimation of the CO_2_/N_2_ selectivity by ML compared to the experimental results. The ML-predicted and simulated CO_2_/N_2_ selectivities (8.8 and 7.9, respectively) of [BMIM][BF_4_]/UiO-66 are lower than the experimental selectivity (14), as shown in Figure 6e. We note that this underestimation does not change our final judgment about the materials’ separation performance because a selectivity on the order of 10 is achieved by both experiments and computations. The results also demonstrate that the experimentally measured CO_2_/N_2_ selectivity of the composite is in the range of mediocre selectivity (8–15), as expected from ML predictions in Figure 5b. We compared the CO_2_/N_2_ selectivity of the [BMIM][BF_4_]/UiO-66 composite with the selectivities of the previously synthesized [BMIM][BF_4_]-based composites [BMIM][BF_4_]/ZIF-8 (7.5 wt %),^34^ [BMIM][BF_4_]/ZIF-8 (28.3 wt %),^17^ [BMIM][BF_4_]/Cu-BTC (30 wt %),^8^ and [BMIM][BF_4_]/Cu-BTC (2.2 wt %).^34^ The new composite showed a comparable/higher separation potential than these previously reported [BMIM][BF_4_]/MOF composites having selectivities of 7.89, 11.60, 13.04, and 15.17 at 1 bar and 298 K.

It is important to note that there are several other composites that exhibit higher CO_2_/N_2_ selectivities than [BMIM][BF_4_]/UiO-66, as shown in Figure 5b. We found that the composites exhibiting CO_2_/N_2_ selectivities of >50 are those with high IL loadings, ≥15 wt %. Using IL amounts beyond a certain loading during the composite synthesis may cause leaching of the IL due to weak molecular interactions with the MOF and/or may exceed the incipient wetness limit of the MOF, resulting in a muddy composite.^7^ Simulations indicated that the maximum number of IL molecules that can be incorporated into a unit cell of UiO-66 is 10. However, when we tried to synthesize a composite having an IL loading on the order of 20 wt % (corresponding to eight IL molecules per unit cell of UiO-66), we observed that this IL loading was very close to, if not exceeding, the incipient wetness limit of UiO-66.^59^ In addition, at such extreme IL loadings, it is highly possible that some of the IL molecules may externally coat the MOF particles rather than entering into the pores. In contrast, our simulations and ML models assume that all of the IL molecules are precisely located inside the MOF cages. Hence, to ensure that the synthesized IL/MOF composite has a structure consistent with the simulations, we targeted a moderate IL loading that is well below the incipient wetness point yet high enough to produce a composite that has distinctly different structural characteristics compared to the previously reported composites having the same IL and MOF combinations.^34^ Therefore, we specifically focused on a relatively lower IL loading of 10 wt % in the computational design of the new composite, which eventually yielded a mediocre selectivity. We reiterate that our idea here is to present a proof-of-concept study that integrates ML, molecular simulations, and experiments rather than to design a material with exceptional gas separation performance.

### 3.4 Further Notes on Our Approach

Our previous results^7,17^ demonstrated that the structural characteristics and their consequences on the gas separation performance of the IL/MOF composites strongly depend on the IL loading. For instance, it was shown that the thermal stability of [BMIM][BF_4_]/ZIF-8 composites varies more than 25 °C with an increase in the IL loading from 4 to 28 wt %. It was also demonstrated that the increase in the IL loading has a strong influence on the gas adsorption characteristics and the consequent separation performance of the composite.^17^ For example, the *Q*_st_ values for CO_2_ and N_2_ were measured as 18, 19, 21, and 29 kJ/mol and 8, 10, 11, and 13 kJ/mol for pristine ZIF-8 and its composites with [BMIM][BF_4_] having IL loadings of 4, 20, and 28 wt %, respectively. Among these composites, the highest difference between the *Q*_st_ values of gases was measured for the composite with 28 wt % IL loading, which also provided the highest CO_2_/N_2_ selectivity. The composite that we synthesized in this work has a distinctly different IL loading (10.4 wt %) than the [BMIM][BF_4_]/UiO-66 composite (3.4 wt %) that we previously presented to validate the molecular simulation methodology, which was introduced to study the [BMIM][BF_4_]/MOF composites.^34^ Hence, we infer that the composite that we presented here is a completely new material because its IL loading is distinctly different from that presented in our previous report.^34^

The accuracy of ML models predicting the gas separation performance of IL/MOF composites is strongly dependent on the assumptions that we implemented in molecular simulations. Therefore, we highlight the main assumptions of the simulations: GCMC simulations overestimate experiments, particularly for N_2_ adsorption of the [BMIM][BF_4_]/UiO-66 composite, because the IL exhibits different behavior in the confined geometry compared to its bulk state. The specific intermolecular interactions between IL molecules and the MOF, which were observed through IR analysis of the [BMIM][BF_4_]/UiO-66 composite in the experimental section and discussed in the SI, were not defined in molecular simulations. A generic force field and an approximate charge assignment method were used for simulating a large number and variety of MOFs and IL/MOF composites, but they may not accurately represent all types of MOFs and ILs.^60^ We reassigned the partial charges of the composite after IL incorporation at least to partially reflect the change in the electronic environment; however, charges only account for calculation of the electrostatic interactions between the composite and gas molecules. Despite these assumptions and considering that the only experimental input to the GCMC simulations is the CIF of UiO-66, there is a reasonably good agreement between the experiments and simulations for the CO_2_ and N_2_ adsorption isotherms of pristine UiO-66, as shown in Figure S10.

Because we aimed to integrate the experimental measurements of pure gas adsorption with the results of molecular simulations and ML models in this proof-of-concept study, we specifically focused on single-component adsorption simulations. However, single-component conditions do not consider the interactions between CO_2_ and N_2_ molecules, which might affect the selectivities of the materials. Therefore, we compared the ideal selectivities of the MOFs and [BMIM][BF_4_]/MOF composites calculated in this work with the mixture selectivities reported in our previous work^34^ in Figure S11. The CO_2_/N_2_ (15/85) mixture selectivities of the IL/MOF composites (2.1–1132.3) and MOFs (1.8–359.9) are higher than the ideal selectivities of the composites (2.2–76.3) and MOFs (1.9–57.6), respectively. This difference in the ideal and mixture selectivities originated from the competition between the gas species for the same adsorption sites, and as a result, strongly adsorbing CO_2_ excludes the weakly adsorbing N_2_. High mixture selectivities indicate the high separation potentials of the [BMIM][BF_4_]/MOF composites and their corresponding MOFs in realistic conditions. We believe that further studies on the different types of MOFs and different [BMIM][BF_4_] loadings may produce new composites offering much higher CO_2_/N_2_ selectivities. Overall, integrating molecular simulations with ML has been an accurate and efficient approach to predicting the gas adsorption and separation performances of IL/MOF composites. Extending this approach to different IL-incorporated porous materials offers broad potential for the rational design of novel materials.

## 4 Conclusion

We performed molecular simulations to investigate almost 1000 [BMIM][BF_4_]/MOF composites for CO_2_ and N_2_ adsorption and then, used the data to develop ML models that can accurately predict the CO_2_ and N_2_ uptakes and CO_2_/N_2_ selectivities of composites. Multivariate analysis based on the ML results revealed the hidden importance of several structural features determining the CO_2_/N_2_ separation performances of [BMIM][BF_4_]/MOF composites. The most important features that affect the CO_2_/N_2_ selectivity were used to computationally generate a new [BMIM][BF_4_]/UiO-66 (10.4 wt %) composite that was not present in our initial composite database. We experimentally synthesized this composite, characterized it in detail, and measured the CO_2_ and N_2_ adsorption properties. The results showed that the CO_2_/N_2_ selectivity of the [BMIM][BF_4_]/UiO-66 composite predicted by the ML models agrees well with the experimentally measured CO_2_/N_2_ selectivity. Overall, we demonstrated an accurate and efficient approach integrating molecular simulations with ML to assess the CO_2_/N_2_ separation performances of any [BMIM][BF_4_]/MOF composite in a time-efficient manner, compared to the extensive time, effort, and cost requirements of the experimental methods.

## Supporting Information Available

Details of molecular simulations, ML algorithms, ML-predicted CO_2_ and N_2_ uptakes for MOFs, and univariate analysis of the MOFs and IL/MOF composites, comparison of the ideal and mixture CO_2_/N_2_ selectivities, details of experiments including materials, sample preparation, characterization, gas adsorption measurements, and washing experiments, and 20 different features of the MOFs and [BMIM][BF_4_]/MOF composites, their pure CO_2_ and N_2_ uptakes used for ML, and all ML models and TPOT parameters that we used, which are available on GitHub (https://github.com/hdaglar/BMIM.BF4.MOF_Composites_ML) (PDF)

## References

[ref1] Siegelman R. L.; Kim E. J.; Long J. R. Porous Materials for Carbon Dioxide Separations. Nat. Mater. 2021, 20 (8), 1060–1072. 10.1038/s41563-021-01054-8. 34321657

[ref2] Trickett C. A.; Helal A.; Al-Maythalony B. A.; Yamani Z. H.; Cordova K. E.; Yaghi O. M. The Chemistry of Metal–Organic Frameworks for CO_2_ Capture, Regeneration and Conversion. Nat. Rev. Mater. 2017, 2 (8), 1–16. 10.1038/natrevmats.2017.45.

[ref3] Wang C.; Liu D.; Lin W. Metal–Organic Frameworks as a Tunable Platform for Designing Functional Molecular Materials. J. Am. Chem. Soc. 2013, 135 (36), 13222–13234. 10.1021/ja308229p. 23944646 PMC3800686

[ref4] Fujie K.; Kitagawa H. Ionic Liquid Transported into Metal–Organic Frameworks. Coord. Chem. Rev. 2016, 307, 382–390. 10.1016/j.ccr.2015.09.003.

[ref5] Durak Ö.; Zeeshan M.; Habib N.; Gülbalkan H. C.; Alsuhile A. A. A. M.; Çağlayan H. P.; Kurtoğlu-Öztulum S. F.; Zhao Y.; Haşlak Z. P.; Keskin S.; Uzun A. Composites of Porous Materials with Ionic Liquids: Synthesis, Characterization, Applications, and Beyond. Microporous Mesoporous Mater. 2022, 332, 111703 10.1016/j.micromeso.2022.111703.

[ref6] Zeeshan M.; Gulbalkan H. C.; Durak O.; Haslak Z. P.; Unal U.; Keskin S.; Uzun A. An Integrated Computational–Experimental Hierarchical Approach for the Rational Design of an IL/UiO-66 Composite Offering Infinite CO_2_ Selectivity. Adv. Funct. Mater. 2022, 32, 2204149 10.1002/adfm.202204149.

[ref7] Sezginel K. B.; Keskin S.; Uzun A. Tuning the Gas Separation Performance of CuBTC by Ionic Liquid Incorporation. Langmuir 2016, 32 (4), 1139–1147. 10.1021/acs.langmuir.5b04123. 26741463

[ref8] Polat H. M.; Zeeshan M.; Uzun A.; Keskin S. Unlocking CO_2_ Separation Performance of Ionic Liquid/CuBTC Composites: Combining Experiments with Molecular Simulations. Chem. Eng. J. 2019, 373, 1179–1189. 10.1016/j.cej.2019.05.113.

[ref9] Zeeshan M.; Gulbalkan H. C.; Haslak Z. P.; Keskin S.; Uzun A. Doubling CO_2_/N_2_ Separation Performance of CuBTC by Incorporation of 1-n-ethyl-3-methylimidazolium diethyl Phosphate. Microporous Mesoporous Mater. 2021, 316, 110947 10.1016/j.micromeso.2021.110947.

[ref10] Kulak H.; Polat H. M.; Kavak S.; Keskin S.; Uzun A. Improving CO_2_ Separation Performance of MIL-53 (Al) by Incorporating 1-n-Butyl-3-Methylimidazolium Methyl Sulfate. Energy Technol. 2019, 7 (7), 1900157 10.1002/ente.201900157. PMC7043311 32140382

[ref11] Kavak S.; Polat H. M.; Kulak H.; Keskin S.; Uzun A. MIL-53 (Al) as a Versatile Platform for Ionic-Liquid/MOF Composites to Enhance CO_2_ Selectivity over CH_4_ and N_2_. Chem. Asian J. 2019, 14 (20), 3655–3667. 10.1002/asia.201900634. 31339661 PMC6851973

[ref12] Oliveira L. T.; Goncalves R. V.; Gonçalves D. V.; de Azevedo D. C. S.; Pereira de Lucena S. M. Superior Performance of Mesoporous MOF MIL-100 (Fe) Impregnated with Ionic Liquids for CO_2_ Adsorption. J. Chem. Eng. Data 2019, 64 (5), 2221–2228. 10.1021/acs.jced.8b01177.

[ref13] Ma J.; Ying Y.; Guo X.; Huang H.; Liu D.; Zhong C. Fabrication of Mixed-Matrix Membrane Containing Metal–Organic Framework Composite with Task-Specific Ionic Liquid for Efficient CO_2_ Separation. J. Mater. Chem. A 2016, 4 (19), 7281–7288. 10.1039/C6TA02611G.

[ref14] Kinik F. P.; Altintas C.; Balci V.; Koyuturk B.; Uzun A.; Keskin S. [BMIM][PF_6_] Incorporation Doubles CO_2_ Selectivity of ZIF-8: Elucidation of Interactions and Their Consequences on Performance. ACS Appl. Mater. Interfaces 2016, 8 (45), 30992–31005. 10.1021/acsami.6b11087. 27783899

[ref15] Ban Y.; Li Z.; Li Y.; Peng Y.; Jin H.; Jiao W.; Guo A.; Wang P.; Yang Q.; Zhong C.; Yang W. Confinement of Ionic Liquids in Nanocages: Tailoring the Molecular Sieving Properties of ZIF-8 for Membrane-based CO_2_ Capture. Angew. Chem., Int. Ed. 2015, 54 (51), 15483–15487. 10.1002/anie.201505508. 26515558

[ref16] Ferreira T. J.; Vera A. T.; De Moura B. A.; Esteves L. M.; Tariq M.; Esperança J. M.; Esteves I. A. Paramagnetic Ionic Liquid/Metal Organic Framework Composites for CO_2_/CH_4_ and CO_2_/N_2_ Separations. Front. Chem. 2020, 8, 590191 10.3389/fchem.2020.590191. 33304882 PMC7701274

[ref17] Koyuturk B.; Altintas C.; Kinik F. P.; Keskin S.; Uzun A. Improving Gas Separation Performance of ZIF-8 by [BMIM][BF_4_] Incorporation: Interactions and Their Consequences on Performance. J. Phys. Chem. C 2017, 121 (19), 10370–10381. 10.1021/acs.jpcc.7b00848.

[ref18] Zeeshan M.; Keskin S.; Uzun A. Enhancing CO_2_/CH_4_ and CO_2_/N_2_ Separation Performances of ZIF-8 by Post-Synthesis Modification with [BMIM][SCN]. Polyhedron 2018, 155, 485–492. 10.1016/j.poly.2018.08.073.

[ref19] Zeeshan M.; Kulak H.; Kavak S.; Polat H. M.; Durak O.; Keskin S.; Uzun A. Influence of Anion Size and Electronic Structure on the Gas Separation Performance of Ionic Liquid/ZIF-8 Composites. Microporous Mesoporous Mater. 2020, 306, 110446 10.1016/j.micromeso.2020.110446.

[ref20] Mohamedali M.; Ibrahim H.; Henni A. Incorporation of Acetate-Based Ionic Liquids into a Zeolitic Imidazolate Framework (ZIF-8) as Efficient Sorbents for Carbon Dioxide Capture. Chem. Eng. J. 2018, 334, 817–828. 10.1016/j.cej.2017.10.104.

[ref21] Philip F. A.; Henni A. Enhancement of Post-Combustion CO_2_ Capture Capacity by Incorporation of Task-Specific Ionic Liquid into ZIF-8. Microporous Mesoporous Mater. 2022, 330, 111580 10.1016/j.micromeso.2021.111580.

[ref22] Hussain S.; Dong H.; Zhang Y.; Zhan G.; Zeng S.; Duan H.; Zhang X. Impregnation of 1-n-Butyl-3-methylimidazolium Dicyanide [BMIM][DCA] into ZIF-8 as a Versatile Sorbent for Efficient and Selective Separation of CO_2_. Ind. Eng. Chem. Res. 2022, 61 (1), 706–715. 10.1021/acs.iecr.1c03798.

[ref23] Ferreira T. J.; Ribeiro R. P.; Mota J. P.; Rebelo L. P.; Esperança J. M.; Esteves I. A. Ionic Liquid-Impregnated Metal–Organic Frameworks for CO_2_/CH_4_ Separation. ACS Appl. Nano Mater. 2019, 2 (12), 7933–7950. 10.1021/acsanm.9b01936.

[ref24] Zeeshan M.; Nozari V.; Yagci M. B.; Isık T.; Unal U.; Ortalan V.; Keskin S.; Uzun A. Core–Shell Type Ionic Liquid/Metal Organic Framework Composite: An Exceptionally High CO_2_/CH_4_ Selectivity. J. Am. Chem. Soc. 2018, 140 (32), 10113–10116. 10.1021/jacs.8b05802. 30005163

[ref25] Gorman J. Faster, Better, Cleaner?: New Liquids Take Aim at Old-Fashioned Chemistry. Science News 2001, 160 (10), 156–158. 10.2307/4012654.

[ref26] The Cambridge Structural Database (CSD). https://www.ccdc.cam.ac.uk/CCDCStats/Stats (accessed 2022-08-26).

[ref27] Chung Y. G.; Haldoupis E.; Bucior B. J.; Haranczyk M.; Lee S.; Zhang H.; Vogiatzis K. D.; Milisavljevic M.; Ling S.; Camp J. S.; et al. Advances, Updates, and Analytics for the Computation-Ready, Experimental Metal–Organic Framework Database: CoRE MOF 2019. J. Chem. Eng. Data 2019, 64 (12), 5985–5998. 10.1021/acs.jced.9b00835.

[ref28] Li A.; Perez R. B.; Wiggin S.; Ward S. C.; Wood P. A.; Fairen-Jimenez D. The Launch of A Freely Accessible MOF CIF Collection from The CSD. Matter 2021, 4 (4), 1105–1106. 10.1016/j.matt.2021.03.006.

[ref29] Tong M.; Lan Y.; Yang Q.; Zhong C. Exploring the Structure-Property Relationships of Covalent Organic Frameworks for Noble Gas Separations. Chem. Eng. Sci. 2017, 168, 456–464. 10.1016/j.ces.2017.05.004.

[ref30] Ongari D.; Yakutovich A. V.; Talirz L.; Smit B. Building a Consistent and Reproducible Database for Adsorption Evaluation in Covalent–Organic Frameworks. ACS Cent. Sci. 2019, 5 (10), 1663–1675. 10.1021/acscentsci.9b00619. 31681834 PMC6822289

[ref31] Colón Y. J.; Gómez-Gualdrón D. A.; Snurr R. Q. Topologically Guided, Automated Construction of Metal–Organic Frameworks and Their Evaluation for Energy-Related Applications. Cryst. Growth Des. 2017, 17 (11), 5801–5810. 10.1021/acs.cgd.7b00848.

[ref32] Wilmer C. E.; Leaf M.; Lee C. Y.; Farha O. K.; Hauser B. G.; Hupp J. T.; Snurr R. Q. Large-Scale Screening of Hypothetical Metal–Organic Frameworks. Nat. Chem. 2012, 4 (2), 83–89. 10.1038/nchem.1192. 22270624

[ref33] Lan Y.; Yan T.; Tong M.; Zhong C. Large-Scale Computational Assembly of Ionic Liquid/MOF Composites: Synergistic Effect in the Wire-Tube Conformation for Efficient CO_2_/CH_4_ Separation. J. Mater. Chem. A 2019, 7 (20), 12556–12564. 10.1039/C9TA01752F.

[ref34] Polat H. M.; Kavak S.; Kulak H.; Uzun A.; Keskin S. CO_2_ Separation from Flue Gas Mixture Using [BMIM][BF_4_]/MOF Composites: Linking High-Throughput Computational Screening with Experiments. Chem. Eng. J. 2020, 394, 124916 10.1016/j.cej.2020.124916.

[ref35] Moghadam P. Z.; Li A.; Wiggin S. B.; Tao A.; Maloney A. G.; Wood P. A.; Ward S. C.; Fairen-Jimenez D. Development of a Cambridge Structural Database subset: a collection of metal–organic frameworks for past, present, and future. Chem. Mater. 2017, 29 (7), 2618–2625. 10.1021/acs.chemmater.7b00441.

[ref36] Frisch M.; Trucks G.; Schlegel H.; Scuseria G.; Robb M.; Cheeseman J.; Scalmani G.; Barone V.; Petersson G.; Nakatsuji H. Gaussian16; Gaussian, Inc.: Wallingford, CT, 2016.

[ref37] Raghavachari K. Perspective on “Density Functional Thermochemistry. III. The Role of Exact Exchange. Theor. Chem. Acc. 2000, 103 (3), 361–363. 10.1007/978-3-662-10421-7_60.

[ref38] Lee C.; Yang W.; Parr R. G. Development of the Colle-Salvetti Correlation-Energy Formula into A Functional of the Electron Density. Phys. Rev. B 1988, 37 (2), 785 10.1103/PhysRevB.37.785. 9944570

[ref39] Baker J. An Algorithm for the Location of Transition States. J. Comput. Chem. 1986, 7 (4), 385–395. 10.1002/jcc.540070402.

[ref40] Dubbeldam D.; Calero S.; Ellis D. E.; Snurr R. Q. RASPA: Molecular Simulation Software for Adsorption and Diffusion in Flexible Nanoporous Materials. Mol. Simul. 2016, 42 (2), 81–101. 10.1080/08927022.2015.1010082.

[ref41] Foster a. J.; Weinhold F. Natural Hybrid Orbitals. J. Am. Chem. Soc. 1980, 102 (24), 7211–7218. 10.1021/ja00544a007.

[ref42] Reed A. E.; Curtiss L. A.; Weinhold F. Intermolecular Interactions From a Natural Bond Orbital, Donor-Acceptor Viewpoint. Chem. Rev. 1988, 88 (6), 899–926. 10.1021/cr00088a005.

[ref43] Frenkel D.; Smit B. Understanding Molecular Simulation: From Algorithms to Applications; Elsevier, 2001; Vol. 1.

[ref44] Willems T. F.; Rycroft C. H.; Kazi M.; Meza J. C.; Haranczyk M. Algorithms and Tools for High-Throughput Geometry-Based Analysis of Crystalline Porous Materials. Microporous Mesoporous Mater. 2012, 149 (1), 134–141. 10.1016/j.micromeso.2011.08.020.

[ref45] Olson R. S.; Urbanowicz R. J.; Andrews P. C.; Lavender N. A.; Kidd L. C.; Moore J. H. Automating Biomedical Data Science Through Tree-Based Pipeline Optimization, European Conference on the Applications of Evolutionary Computation; Springer, 2016; pp 123–137.

[ref46] Orhan I. B.; Daglar H.; Keskin S.; Le T. C.; Babarao R. Prediction of O_2_/N_2_ Selectivity in Metal–Organic Frameworks via High-Throughput Computational Screening and Machine Learning. ACS Appl. Mater. Interfaces 2022, 14, 736–749. 10.1021/acsami.1c18521. 34928569

[ref47] Tang H.; Xu Q.; Wang M.; Jiang J. Rapid Screening of Metal–Organic Frameworks for Propane/Propylene Separation by Synergizing Molecular Simulation and Machine Learning. ACS Appl. Mater. Interfaces 2021, 13 (45), 53454–53467. 10.1021/acsami.1c13786. 34665615

[ref48] Daglar H.; Keskin S. Combining Machine Learning and Molecular Simulations to Unlock Gas Separation Potentials of MOF Membranes and MOF/Polymer MMMs. ACS Appl. Mater. Interfaces 2022, 14, 32134–32148. 10.1021/acsami.2c08977. 35818710 PMC9305976

[ref49] Dureckova H.; Krykunov M.; Aghaji M. Z.; Woo T. K. Robust Machine Learning Models for Predicting High CO_2_ Working Capacity and CO_2_/H_2_ Selectivity of Gas Adsorption in Metal Organic Frameworks for Precombustion Carbon Capture. J. Phys. Chem. C 2019, 123 (7), 4133–4139. 10.1021/acs.jpcc.8b10644.

[ref50] Liang H.; Jiang K.; Yan T.-A.; Chen G.-H. XGBoost: An Optimal Machine Learning Model with Just Structural Features to Discover MOF Adsorbents of Xe/Kr. ACS Omega 2021, 6 (13), 9066–9076. 10.1021/acsomega.1c00100. 33842776 PMC8028164

[ref51] Habib N.; Durak O.; Zeeshan M.; Uzun A.; Keskin S. A Novel IL/MOF/Polymer Mixed Matrix Membrane Having Superior CO_2_/N_2_ Selectivity. J. Membr. Sci. 2022, 658, 120712 10.1016/j.memsci.2022.120712.

[ref52] Zhang H.; Nettleton D.; Zhu Z. Regression-Enhanced Random Forests. arXiv Preprint arXiv:1904.10416, 2019.

[ref53] Groves J. A.; Miller S. R.; Warrender S. J.; Mellot-Draznieks C.; Lightfoot P.; Wright P. A. The First Route to Large Pore Metal Phosphonates. Chem. Commun. 2006, (31), 3305–3307. 10.1039/b605400e. 16883418

[ref54] Huang S.-L.; Jia A.-Q.; Jin G.-X. Pd(diimine)Cl_2_ Embedded Heterometallic Compounds with Porous Structures as Efficient Heterogeneous Catalysts. Chem. Commun. 2013, 49 (24), 2403–2405. 10.1039/c3cc38714c. 23417107

[ref55] Chen X. D.; Wan C. Q.; Sung H. H.-Y.; Williams I. D.; Mak T. C. Control of Channel Size for Selective Guest Inclusion with Inlaid Anionic Building Blocks in a Porous Cationic Metal–Organic Host Framework. Chem. Eur. J. 2009, 15 (26), 6518–6528. 10.1002/chem.200900010. 19449359

[ref56] Park J.; Howe J. D.; Sholl D. S. How Reproducible are Isotherm Measurements in Metal–Organic Frameworks?. Chem. Mater. 2017, 29 (24), 10487–10495. 10.1021/acs.chemmater.7b04287.

[ref57] Cavka J. H.; Jakobsen S.; Olsbye U.; Guillou N.; Lamberti C.; Bordiga S.; Lillerud K. P. A Mew Zirconium Inorganic Building Brick Forming Metal Organic Frameworks with Exceptional Stability. J. Am. Chem. Soc. 2008, 130 (42), 13850–13851. 10.1021/ja8057953. 18817383

[ref58] Moghadam P. Z.; Fairen-Jimenez D.; Snurr R. Q. Efficient Identification of Hydrophobic MOFs: Application in the Capture of Toxic Industrial Chemicals. J. Mater. Chem. A 2016, 4 (2), 529–536. 10.1039/C5TA06472D.

[ref59] Durak Ö.; Kulak H.; Kavak S.; Polat H. M.; Keskin S.; Uzun A. Towards Complete Elucidation of Structural Factors Controlling Thermal Stability of IL/MOF Composites: Effects of Ligand Functionalization on MOFs. J. Phys.: Condens. Matter 2020, 32 (48), 484001 10.1088/1361-648X/aba06c. 32590364

[ref60] Singh M. P.; Singh R. K.; Chandra S. Ionic Liquids Confined in Porous Matrices: Physicochemical Properties and Applications. Prog. Mater. Sci. 2014, 64, 73–120. 10.1016/j.pmatsci.2014.03.001.

