# Supplementary material for: Integrating Molecular Simulations with Machine Learning Guides in the Design and Synthesis of [BMIM][BF4]/MOF Composites for CO2/N2 Separation
Source: ACS Appl Mater Interfaces. 2023 Mar 27;15(13):17421–31. doi: 10.1021/acsami.3c02130 (PMC10080536; doi:10.1021/acsami.3c02130)
Supplement: Supplementary file 1 — am3c02130_si_001.pdf [file am3c02130_si_001.pdf]

Supporting Information

Integrating Molecular Simulations with Machine Learning Guides the Design and Synthesis of [BMIM][BF$_4$]/MOF Composites for CO_2/N_2 Separation

Hilal Daglar,[a] Hasan Can Gulbalkan,[a,†] Nitasha Habib,[a,b,†] Ozce Durak,[a,b] Alper Uzun[*,a,b,c] and Seda Keskin[*,a]

[a]Department of Chemical and Biological Engineering, Koç University, Rumelifeneri Yolu, Sariyer, 34450, Istanbul, Turkey

[b]Koç University TÜPRAŞ Energy Center (KUTEM), Koç University, Rumelifeneri Yolu, 34450 Sariyer, Istanbul, Turkey

[c]Koç University Surface Science and Technology Center (KUYTAM), Koç University, Rumelifeneri Yolu, 34450 Sariyer, Istanbul, Turkey

Submitted to *ACS Applied Materials & Interfaces*

*Corresponding authors: e-mail: skeskin@ku.edu.tr, auzun@ku.edu.tr
Phone: +90 (212) 338-1362
†These authors contributed equally.

Table of Contents

1. Previously synthesized IL/MOF composites for CO_2 separations 3
2. Methods 4
2.1 Molecular Simulations and Selectivity Calculations 4
2.3 Machine Learning 5
3. Experimental Methods 11
Materials. 11
Sample preparation. 11
Brunauer-Emmer-Teller (BET) surface area and t-plot pore volume analysis 12
X-ray diffraction (XRD) spectroscopy. 12
Scanning electron microscopy (SEM). 12
Energy dispersive X-ray (EDX) spectroscopy. 12
Fourier transform infrared (FTIR) spectroscopy 13
4. Characterizations of UiO-66 and [BMIM][BF_4]/UiO-66 13
High-pressure volumetric gas adsorption measurements. 18

1. Previously synthesized IL/MOF composites for CO_2 separations

Table S1 shows the experimentally synthesized IL/MOF composites studied for CO_2/N_2, CO_2/CH_4, and CH_4/N_2 separations in the literature.

Table S1. Previously synthesized IL/MOF composites for CO_2/N_2, CO_2/CH_4, and CH_4/N_2 separations

IL/MOF Composite	Separation	Ref.
[BMPyrr][DCA]/UiO-66	CO_2/N_2, CO_2/CH_4, CH_4/N_2	1
[BMIM][BF_4]/Cu-BTC	CO_2/N_2, CO_2/CH_4, CH_4/N_2	2
[BMIM][CF_3SO_3]/Cu-BTC	CO_2/N_2, CO_2/CH_4, CH_4/N_2	3
[BMIM][$MeSO_4$]/Cu-BTC	CO_2/N_2, CO_2/CH_4, CH_4/N_2	3
[BMIM][$MeSO_3$]/Cu-BTC	CO_2/N_2, CO_2/CH_4, CH_4/N_2	3
[BMIM][SbF_6]/Cu-BTC	CO_2/N_2, CO_2/CH_4, CH_4/N_2	3
[BMIM][PF_6]/Cu-BTC	CO_2/N_2, CO_2/CH_4, CH_4/N_2	3
[BMIM][$OcSO_4$]/Cu-BTC	CO_2/N_2, CO_2/CH_4, CH_4/N_2	3
[BMIM][DCA]/Cu-BTC	CO_2/N_2, CO_2/CH_4, CH_4/N_2	3
[BMIM][DBP]/Cu-BTC	CO_2/N_2, CO_2/CH_4, CH_4/N_2	3
[EMIM][DEP]/Cu-BTC	CO_2/N_2, CO_2/CH_4	4
[BMIM][PF_6]/MIL-100(Fe)	CO_2/N_2, CO_2/CH_4	5
[BMIM][NTf_2]/MIL-100(Fe)	CO_2/N_2, CO_2/CH_4	5
[BMIM][$MeSO_4$]/MIL-53(Al)	CO_2/N_2, CO_2/CH_4	6
[BMIM][NTf_2]/MIL-53(Al)	CO_2/N_2, CO_2/CH_4	7
[BMIM][SbF_6]/MIL-53(Al)	CO_2/N_2, CO_2/CH_4	7
[BMIM][CF_3SO_3]/MIL-53(Al)	CO_2/N_2, CO_2/CH_4	7
[BMIM][PF_6]/MIL-53(Al)	CO_2/N_2, CO_2/CH_4	7
[BMIM][BF_4]/MIL-53(Al)	CO_2/N_2, CO_2/CH_4	7
[C_3NH_2bim][Tf_2N]/NH_2-MIL-101(Cr)	CO_2/N_2	8
[BMIM][PF_6]/ZIF-8	CO_2/N_2, CO_2/CH_4, CH_4/N_2	9
[C_2MIM][NTf_2]/ZIF-8	CO_2/CH_4	10
[C_2OHMIM][NTf_2]/ZIF-8	CO_2/CH_4	10
[C_6MIM][NTf_2]/ZIF-8	CO_2/CH_4	10
[C_{10}MIM][NTf_2]/ZIF-8	CO_2/CH_4	10
[BzMIM][NTf_2]/ZIF-8	CO_2/CH_4	10
[$P_{6,6,6,14}$][NTf_2]/ZIF-8	CO_2/CH_4	10
[C_6MIM][$N(CN)_2$]/ZIF-8	CO_2/CH_4	10
[C_6MIM][$C(CN)_3$]/ZIF-8	CO_2/CH_4	10
[C_6MIM][Cl]/ZIF-8	CO_2/CH_4	10
[C_2MIM][Ac]/ZIF-8	CO_2/CH_4	10
[HEMIM][DCA]/ZIF-8	CO_2/CH_4	11
[BMIM][NTf_2]/ZIF-8	CO_2/N_2, CO_2/CH_4	12
[C_4MIM]$_2$[$NiCl_4$]/ZIF-8	CO_2/N_2, CO_2/CH_4	13
[C_4MIM]$_2$[$MnCl_4$]/ZIF-8	CO_2/N_2, CO_2/CH_4	13
[C_4MIM]$_2$[$CoCl_4$]/ZIF-8	CO_2/N_2, CO_2/CH_4	13
[C_4MIM]$_2$[$Co(NCS)_4$]/ZIF-8	CO_2/N_2, CO_2/CH_4	13
[C_4MIM][$FeCl_4$]/ZIF-8	CO_2/N_2, CO_2/CH_4	13
[BMIM][BF_4]/ZIF-8	CO_2/N_2, CO_2/CH_4, CH_4/N_2	14
[BMIM][$MeSO_3$]/ZIF-8	CO_2/N_2, CO_2/CH_4, CH_4/N_2	15
[BMIM][CF_3SO_3]/ZIF-8	CO_2/N_2, CO_2/CH_4, CH_4/N_2	15
[BMIM][$MeSO_4$]/ZIF-8	CO_2/N_2, CO_2/CH_4, CH_4/N_2	15
[BMIM][$OcSO_4$]/ZIF-8	CO_2/N_2, CO_2/CH_4, CH_4/N_2	15
[BMIM][SCN]/ZIF-8	CO_2/N_2, CO_2/CH_4	16
[BMIM][DCA]/ZIF-8	CO_2/CH_4	17
[BMIM][Ac]/ZIF-8	CO_2/N_2	18
[EMIM][Ac]/ZIF-8	CO_2/N_2	18
[EMIM][Gly]/ZIF-8	CO_2/N_2	19
[EMIM][Ala]/ZIF-8	CO_2/N_2	19

2. Methods

2.1 Molecular Simulations and Selectivity Calculations

We focused on the Cambridge Structural Database (CSD) non-disordered MOF subset[20] consisting of 3816 different structures.[21] We then used a Python script from the literature to clean the solvent molecules from the structures.[20] MOFs having a pore limiting diameter (PLD) >6 Å and N_2 accessible surface area (ASA) >0 m^2/g were selected to ensure that IL incorporation into MOFs can be achieved and both CO_2 and N_2 can be adsorbed in the pores of MOFs and IL/MOF composites. The charge equilibration (Qeq)[22] method was used to assign partial charges to MOF atoms to calculate electrostatic interactions between gas molecules and MOF atoms. We removed the MOFs for which atoms have unrealistic partial charges, >+4 or <-2. After these eliminations, 941 MOFs remained.

The IL loadings (wt.%) of [BMIM][BF_4]/MOF composites were calculated as follows; wt.% IL loading= $(N_{[BMIM][BF_4]} \times (w_{[BMIM][BF_4]}/N_A))/(N_{[BMIM][BF_4]} \times (w_{[BMIM][BF_4]}/N_A)) + \rho_{MOF} \times V_{UC}$, where $N_{[BMIM][BF_4]}$ is the number of IL molecules per unit cell (1), $w_{[BMIM][BF_4]}$ is the molar mass of [BMIM][BF_4] (226.02 g/mol), N_A is Avogadro's number (6.022×10^{23}), ρ_{MOF} is the theoretical crystal density of the MOF and V_{UC} is the unit cell volume of the MOF.

Since [BMIM][BF_4] incorporation changes the electronic environment, partial charges of [BMIM][BF_4]/MOF composites were reassigned using the Qeq method to calculate the electrostatic interactions between CO_2, N_2, and the atoms of IL/MOF composites. The van der Waals interactions between gas-gas and gas-materials were defined using Lennard Jones (LJ) potential. The cut-off distance was set to 12 Å for the truncation of non-bonded interactions described by LJ 12-6 potential.[23] Long-range electrostatic interactions were calculated using the Ewald summation.[24] The Dreiding force field[25] was used to obtain the potential parameters for the van der Waals interactions for both MOF and IL atoms. CO_2 was modeled as a three-site rigid molecule with 12-6 LJ potential.[26] N_2 was modeled as a three-site rigid molecule with N atoms at the two ends and a center of mass with a partial point charge as the third site.[27] The Lorentz-Berthelot mixing rules were used to obtain pair potentials between different atoms.

GCMC simulations were performed with 10,000 cycles for initialization and 20,000 cycles for taking the ensemble averages. Translation, rotation, reinsertion, regrow and swap moves were utilized in GCMC simulations for calculating single-component gas uptakes. By using the single-component CO_2 and N_2 uptakes (N_{CO_2} and N_{N_2}, respectively), we calculated the ideal selectivity of MOFs and IL/MOF composites from the formulas $S^{MOF}_{CO_2/N_2} = N^{MOF}_{CO_2}/N^{MOF}_{N_2}$ and $S^{IL/MOF}_{CO_2/N_2} = N^{IL/MOF}_{CO_2}/N^{IL/MOF}_{N_2}$, respectively.

2.3 Machine Learning

We developed ML algorithms predicting CO_2 and N_2 uptake data of MOFs and IL/MOF composites at 1 bar and 298 K using easily obtainable descriptors of materials. **Table S2** shows the 20 features of MOFs and IL/MOF composites that we considered as input data in ML models. We used textural (PLD, LCD), chemical (degree of unsaturation, oxygen-to-metal ratio), and energy-based descriptors (heat of adsorption). Structural properties of pristine MOFs and IL/MOF composites such as PLD, LCD, ASA, geometric pore volume (PV), porosity (ϕ), and density (ρ) were computed using Zeo++ software.[28] The chemical descriptors were obtained from the crystal information file (CIF) of MOFs. We also used the heat of adsorption values (Q^0_{st}) of gases as energy-based descriptors. More details about the selection of features were given in our previous study.[29] We used the Pearson correlation coefficient (r) to determine the feature correlations which can be expressed as, $r=\left(\sum_{i=1}^{n}(x_i-\bar{x})(y_i-\bar{y})\right)/\left(\sqrt{\sum_{i=1}^{n}(x_i-\bar{x})^2}\sqrt{\sum_{i=1}^{n}(y_i-\bar{y})^2}\right)$ where x and y are features, and $\bar{x}$ and $\bar{y}$ are the means of x and y. To avoid multicollinearity problems of features, we only considered the features that do not have a strong correlation with each other ($r<0.9$).[30]

Table S2. Descriptors used to construct a feature vector for ML models.

Feature (Unit)	Symbol
Largest Cavity Diameter (Å)	LCD
Pore limiting diameter (Å)	PLD
Pore size ratio	LCD/PLD
Accessible Surface Area* (m^2/g)	ASA
Density (g/cm^3)	ρ
Pore volume (cm^3/g)	PV
Porosity	φ
IL loading percentage (wt.%)	IL%
Carbon percentage	C%
Hydrogen percentage	H%
Nitrogen percentage	N%
Oxygen percentage	O%
Halogen (Br, Cl, F, I) percentage	Halogen%
Metalloids (As, B, Ge, Te, Sb, Si) percentage	Metalloid%
Ametal (Se, S, P) percentage	Ametal%
Metal percentage	Metal%
Total degree of unsaturation	TDU
Degree of unsaturation	DU
Metallic ratio (# of metal /# of C atoms)	M-to-C
Oxygen to metal ratio	O-to-M
Heat of adsorption (kJ/mol)	Q^0_{st}

* As shown in Figure 2a, ASA is neglected to avoid multicollinearity problems in ML models.

We then used features of materials as input data, CO_2 and N_2 uptakes of materials as target data to train ML models for CO_2/N_2 separation of MOFs and IL/MOF composites at 1 bar, and 298 K. The parameters to find the best algorithms with optimum hyperparameters were listed on GitHub (https://github.com/hdaglar/BMIM.BF4.MOF_Composites_ML). All the regression algorithms we considered were implemented in the scikit-learn toolkit[31] in Python. We used 5-fold cross-validation to avoid overfitting. In ML models, stratified sampling methods were used to split the training and test set having feature distributions as similar as possible.

Table S3. The ML pipelines and parameters based on the gas adsorption properties of MOFs and IL/MOF composites.

Property	Best Pipeline with Parameters
CO_2 adsorption (MOF)	**ExtraTreesRegressor**(MinMaxScaler(), StackingEstimator(estimator=GradientBoostingRegressor(alpha=0.99, learning_rate=0.1, loss="huber", max_depth=8, max_features=0.7000000000000001, min_samples_leaf=14, min_samples_split=16, n_estimators=100, subsample=0.45)),bootstrap=False, max_features=0.55, min_samples_leaf=2, min_samples_split=5, n_estimators=100)
N_2 adsorption (MOF)	**RandomForestRegressor**(StackingEstimator(estimator=RidgeCV()), StackingEstimator(estimator=ExtraTreesRegressor(bootstrap=False, max_features=0.45, min_samples_leaf=5, min_samples_split=10, n_estimators=100)), bootstrap=False, max_features=0.35000000000000003, min_samples_leaf=3, min_samples_split=9, n_estimators=100)
CO_2 adsorption (IL/MOF)	**XGBRegressor** (StandardScaler(), learning_rate=0.1, max_depth=9, min_child_weight=14, n_estimators=100, n_jobs=1, objective="reg:squarederror", subsample=0.6500000000000001, verbosity=0)
N_2 adsorption (IL/MOF)	**XGBRegressor** (PolynomialFeatures(degree=2, include_bias=False, interaction_only=False), learning_rate=0.1, max_depth=9, min_child_weight=13, n_estimators=100, n_jobs=1, objective="reg:squarederror", subsample=0.9500000000000001, verbosity=0)

For the ML model development, the best algorithms were selected based on the quantities related to the model accuracy, such as coefficient of determination (R^2), root mean square error (RMSE), and mean absolute error (MAE) as follows; $R^2=1-\frac{1}{M}\sum_{m=1}^{M}(\bar{y}-\hat{y})^2 / \frac{1}{M}\sum_{m=1}^{M}(y-\hat{y})^2$, $MAE=\sum_{m=1}^{M}|y-\hat{y}|/M$, $RMSE=\sqrt{\sum_{m=1}^{M}(y-\hat{y})^2/M}$. Here, M represents the number of samples, y and $\hat{y}$ represent the simulated (true) value and predicted value, respectively and $\bar{y}$ denotes the average of the simulated value by the model. To demonstrate the materials ranking similarity between ML-predicted and simulated gas adsorption data, we used the Spearman rank correlation coefficient (SRCC) as follows; $SRCC=1-\left(6\sum D^2\right)/\left(n\left(n^2-1\right)\right)$. Here, D represents the difference between paired ranks, and n is the number of observations. ML models with the

highest R^2 and SRCC and the lowest MAE and RMSE are expected to be the best algorithms to predict the target data. Thus, based on R^2, SRCC, MAE, and RMSE, we found the best ML models for CO_2 and N_2 adsorption properties of MOFs and IL/MOF composites.

2.3.1 Univariate Analysis of MOFs and IL/MOF composites

We analyzed the structure-performance relationships between material properties and their CO_2 and N_2 uptakes. We focused on the following features: LCD and PLD as pore-size descriptors, PV and porosity as pore-geometry descriptors, total degree of unsaturation and O-to-M ratio as chemical descriptors, and isosteric heat of adsorption of gases as energy-based descriptors. **Figure S1** shows the relations between these features of IL/MOF composites and the simulated CO_2 and N_2 uptake data. **Figure S1a** shows that the CO_2 uptake of IL/MOF composites decreases as pore size-based descriptors generally increase. As shown in **Figure S1b**, the medium pore volume (0.4-1 g/cm^3) and porosity (<0.6) favor the CO_2 uptake of IL/MOF composites. The highest CO_2 uptakes were observed in the range of 40-55% and 2-8% for C% and Metal%, respectively, as demonstrated in **Figure S1c**. While IL/MOF composites with a high value of CO_2 uptake are in the range of low TDU and O-to-M, this range also has IL/MOF composites exhibiting very low CO_2 uptake, as represented in **Figure S1d**. **Figure S1e** shows that CO_2 uptakes of IL/MOF composites generally increase with increased heat of adsorption at infinite dilution. However, in the medium range ($30<Q^0_{st,\ CO_2}<40$ kJ/mol), many exceptions exist. **Figure S1e** also shows a weak relation between the IL loading of composites and CO_2 uptake. For N_2, **Figure S1f** illustrates that although the narrow pore sizes lead to high gas uptake similar to CO_2, large pore sizes also favor N_2 uptake of IL/MOF composites. There is generally a linear relationship between pore geometry-based descriptors (PV and porosity) and N_2 uptake as represented in **Figure S1g**. This is a sign that N_2 adsorption depends more on space availability in the pores of MOFs than CO_2 adsorption. **Figures S1h-j** show the weak relations between the features of IL/MOF composites (chemical descriptors, Metal%, C%, TDU and O-to-M, IL%, Q^0_{st}) and N_2 uptakes, which suggests that multivariate analysis is required to understand the importance of these features in determining the gas uptake performances of structures. Similar observations are valid for the CO_2 and N_2 uptakes of MOFs, as shown in **Figures S2a-j**.

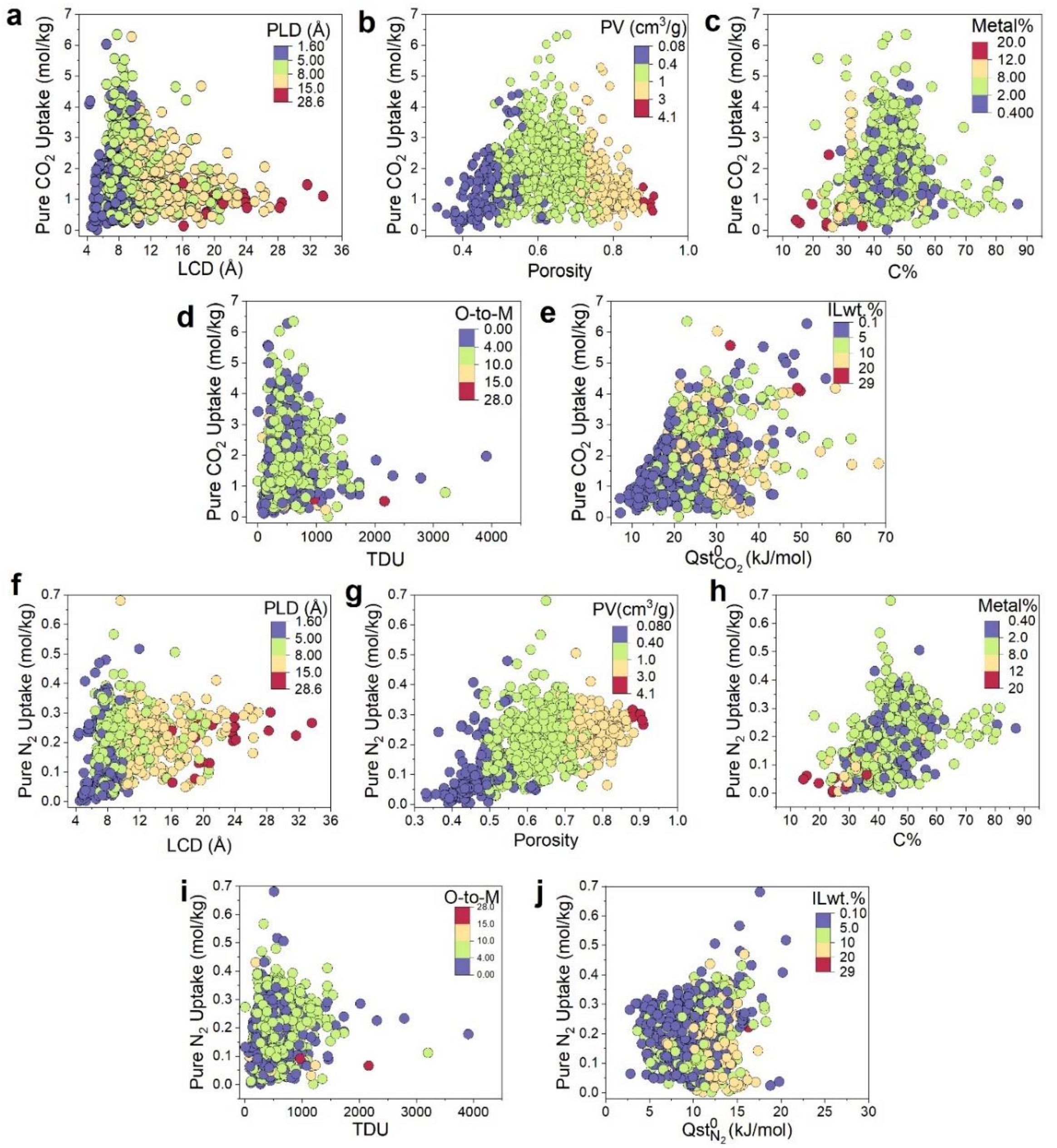

Figure S1. The effect of features on gas uptakes of [BMIM][BF₄]/MOF composites. Simulated CO_2 and N_2 uptakes in 941 [BMIM][BF_4]/MOF composites as a function of **(a, f)** pore size (LCD, PLD), **(b, g)** pore geometry (density, pore volume), **(c, h)** atom types, **(d, i)** chemical descriptors (O-to-M, TDU) and **(e, j)** Q_{st}^0 and IL wt.%.

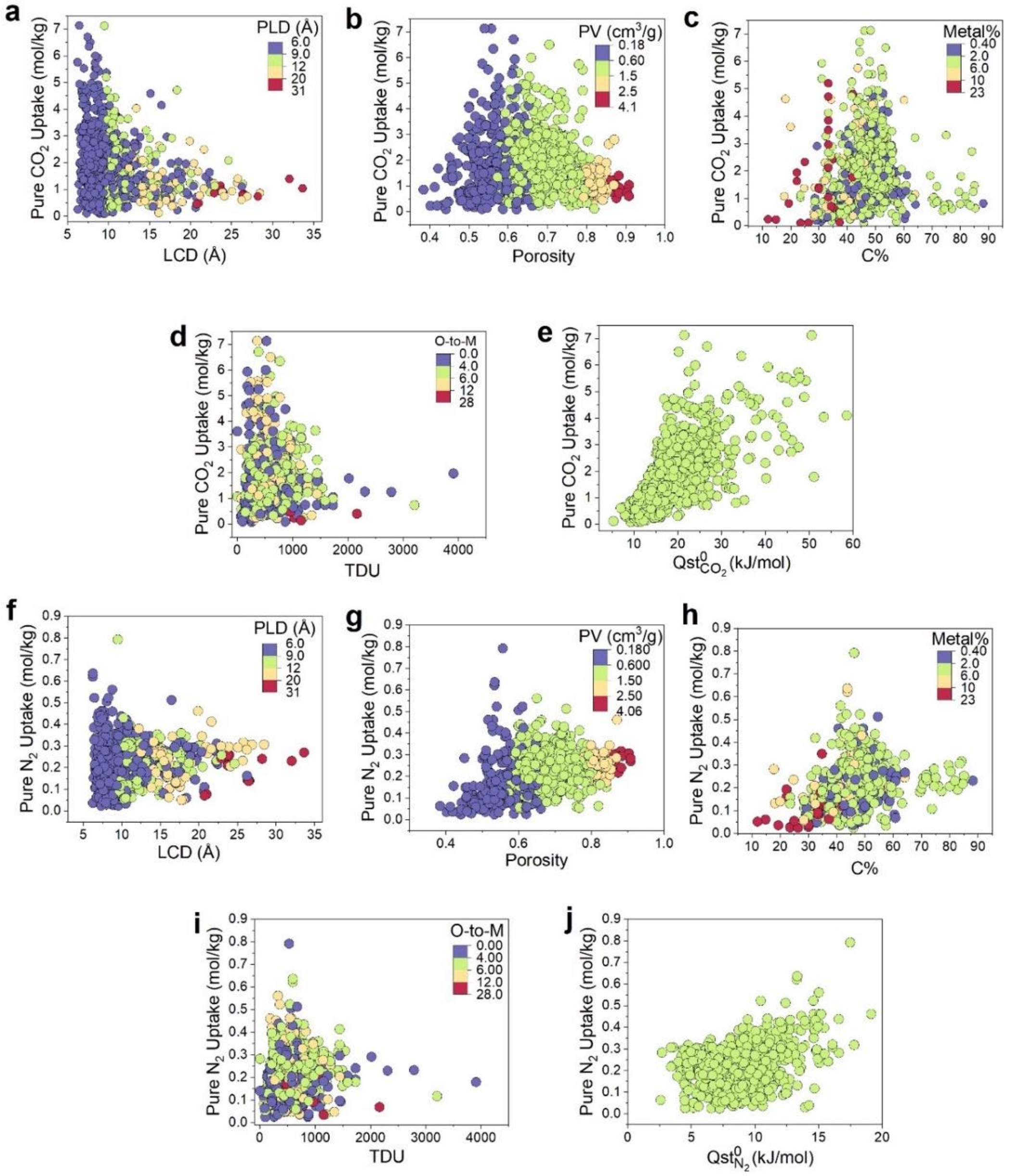

Figure S2. The effect of features on gas uptakes of MOFs. Simulated CO_2 and N_2 uptakes in 941 MOFs as a function of **(a, f)** pore size (LCD, PLD), **(b, g)** pore geometry (density, pore volume), **(c, h)** atom types, **(d, i)** chemical descriptors (O-to-M, TDU) and **(e, j)** Q_{st}^{0}.

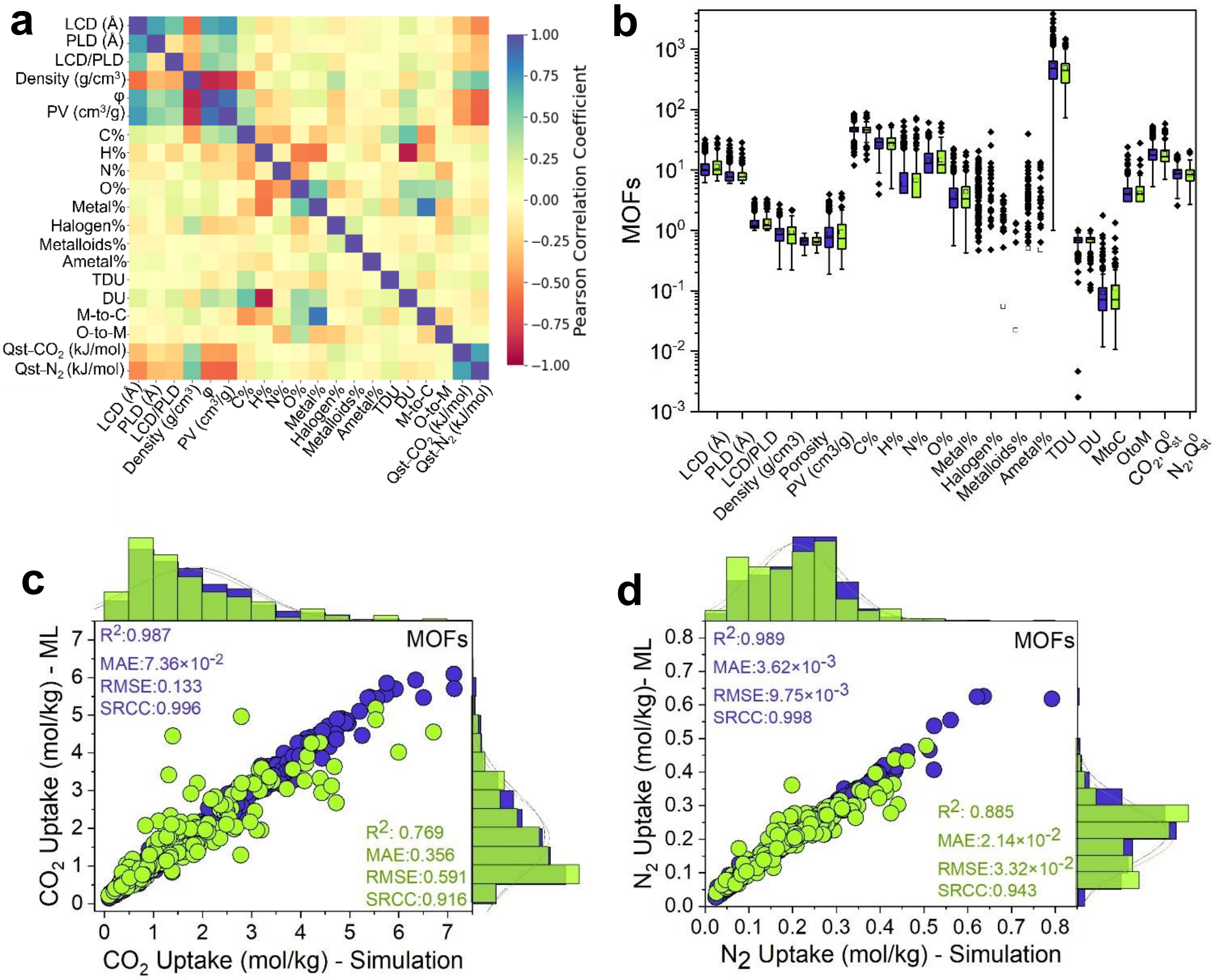

Figure S3. ML models for MOFs. (a) The correlation heatmap of all MOF features. **(b)** Box plots showing the distribution of features based on the training and test set for MOFs. Comparison of ML predictions with the GCMC results for **(c)** CO_2 and **(d)** N_2 uptake of MOFs at 1 bar, 298 K. Marginal histograms show the distribution of gas uptake data in the training and test sets. Training and test sets are represented as purple and green symbols, respectively, in **(b-d)**.

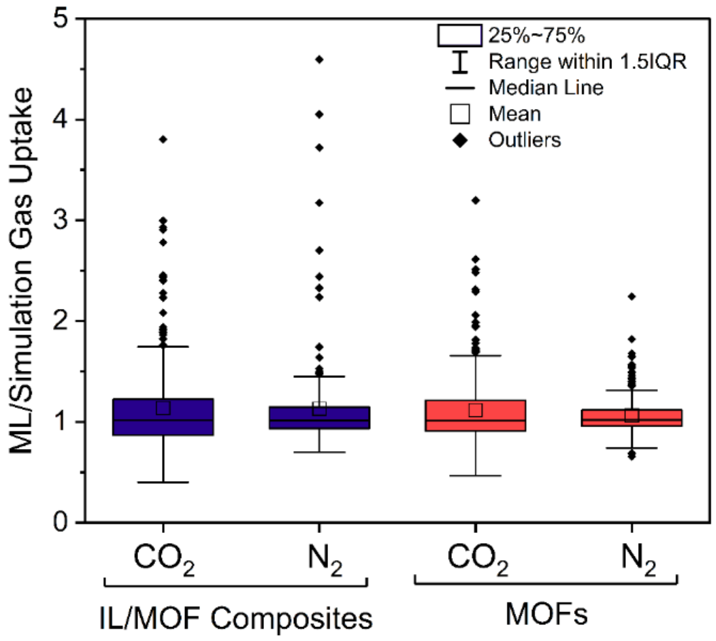

Figure S4. The ratio of ML-predicted uptake to the simulated ones for CO_2 and N_2. Blue and red boxes represent the test set of ML models for IL/MOF composites and MOFs. Boxes show the quartiles of the dataset, while whiskers extend to show the rest of the distribution, except for points that are determined to be outliers. Outliers are defined as values more than 1.5IQR (IQR = interquartile range) from either end of the box.

Table S4. Top three MOFs exhibiting the highest CO_2/N_2 selectivity in the test set.

MOF	CO_2 Uptake (mol/kg) Simulation	CO_2 Uptake (mol/kg) ML	N_2 Uptake (mol/kg) Simulation	N_2 Uptake (mol/kg) ML	CO_2/N_2 Selectivity Simulation	CO_2/N_2 Selectivity ML
QOVDEL	3.84	3.57	0.077	0.088	49.68	40.80
REQBAS	4.10	3.73	0.108	0.104	38.00	35.89
FUSMEM	2.50	2.44	0.066	0.068	37.66	36.16

3. Experimental Methods

Materials. All reagents were purchased from commercial vendors. UiO-66 was purchased from ACSYNAM, Canada. 1-butyl-3-methylimidazolium tetrafluoroborate, [BMIM][BF_4], was purchased from Iolitec and stored in an Argon filled Braun glovebox. Acetone (≥99.5%, reagent grade) was purchased from Sigma-Aldrich. Gases, CO_2 (99.9 vol.%) and N_2 (99.9 vol.%), used in gas adsorption measurements were purchased from Air Liquide.

Sample preparation. Prior to the composite synthesis, pristine UiO-66 was pre-treated overnight at 150 °C under vacuum. For 10.4 wt.% IL loading, 0.104 g of [BMIM][BF_4] was

dissolved in approximately 20 ml acetone for 1-h under constant mixing at 250 rpm. Then, approximately 0.896 g of pristine UiO-66 was added into the [BMIM][BF_4]-acetone solution. Resulting mixture having IL-acetone-MOF was kept under stirring at 35°C in an open atmosphere for 6 h to completely evaporate the solvent. The resulting powder was dried overnight in the oven at 105 °C to evaporate the remaining solvent. The final product, [BMIM][BF_4]/UiO-66 composite, was placed inside the Argon filled glovebox.

Brunauer-Emmer-Teller (BET) surface area and t-plot pore volume analysis. Surface area analysis of pristine UiO-66 and [BMIM][BF_4]/UiO-66 composite was performed by using a Micromeritics ASAP 2020 physisorption analyzer. N_2 adsorption and desorption data obtained at -196 °C were analyzed both for surface area and pore volume. Prior to the measurement, each sample was activated by degassing overnight at 150 °C for 12 h. After the activation step, samples were cooled down to -196 °C with the help of liquid nitrogen, and free space measurements were performed using He gas. Later, N_2 adsorption and desorption isotherms were obtained between a pressure range of 10^{-3} and 1 bar. Pressure steps between 0.05 and 0.3 bar were fitted to the BET equation, and t-plot analysis was conducted. Obtained data were used to determine the surface area and pore volumes of each sample.

X-ray diffraction (XRD) spectroscopy. XRD analysis of pristine UiO-66 and [BMIM][BF_4]/ UiO-66 composite was performed by using a Bruker D2 Phaser instrument with a Lynxeye detector. For X-ray generator, Cu-Kα_1 radiation source with a wavelength of 1.54060 Å was used at 30 kV voltage and 10 mA current. Slit size of 0.2 mm was chosen for Lynxeye detector, whereas the diffraction data was collected between the 2θ range of 5-50° with a step size of 0.0204°. XRD pattern of pristine UiO-66 was simulated by using VESTA software.[32]

Scanning electron microscopy (SEM). SEM images of pristine UiO-66 and [BMIM][BF_4]/UiO-66 composite were obtained by using a Zeiss Evo LS 15 scanning electron microscope. Samples were coated with gold prior to the analysis to prevent charging issues. Analyses were conducted under vacuum with an accelerating voltage of 3 kV and a working distance of 5 mm. SEM images were taken with a magnification of 10 and 50 k×.

Energy dispersive X-ray (EDX) spectroscopy. EDX mapping was conducted by using a Zeiss Evo LS 15 scanning electron microscope equipped with a Bruker XFlash 5010 EDX detector. EDX images were collected with EHT of 5 kV, 123 eV resolution, and a working distance of 6 mm for both pristine material and composite.

Fourier transform infrared (FTIR) spectroscopy. IR spectra were obtained by using a Bruker Vertex 80v IR spectrometer equipped with a platinum attenuated total reflection (ATR) accessory. Data was collected as an average of 256 scans for both the samples and corresponding backgrounds. A spectral resolution of 2 cm^{-1} was used, and the analysis was performed under atmospheric pressure between the wavelength range of 4000-400 cm^{-1}. The resulting IR data was compensated for atmospheric gases, such as H_2O and CO_2, and corrected by using extended ATR modification with the help of OPUS software. Deconvolution of the IR bands was conducted with the help of Fityk software using the Voigt function.[33]

4. Characterizations of UiO-66 and [BMIM][BF$_4$]/UiO-66

The IL loading of the synthesized [BMIM][BF$_4$]/UiO-66 was determined by washing the composite several times with acetone, a solvent having a molecular size of 6 Å that can enter the pore openings of UiO-66 (8 Å).[34] After washing, the filtrate was collected in a vial, and the washed IL/MOF composite was dried overnight at 80 °C. The IR spectra of the IL/MOF composite before and after washing, pristine UiO-66, bulk [BMIM][BF$_4$], the filtrate, and pure acetone are presented in **Figure S4**. The IR spectra of filtrate showed the characteristic peaks of [BMIM][BF$_4$], and the spectra of washed and dried [BMIM][BF$_4$]/UiO-66 composite was identical to that of the pristine UiO-66. Thus, from these observations, it was confirmed that the IL molecules were removed from the cages of the synthesized IL/MOF composite. The IL loading of the composite was determined as approximately 9.2±0.6 wt.%. We note that the measured amount is slightly less than the stoichiometric IL loading of 10.4 wt.% targeted to prepare [BMIM][BF$_4$]/UiO-66 composite. This difference can be attributed to the loss of some IL on the walls of the sample container during the synthesis process of the composite sample.

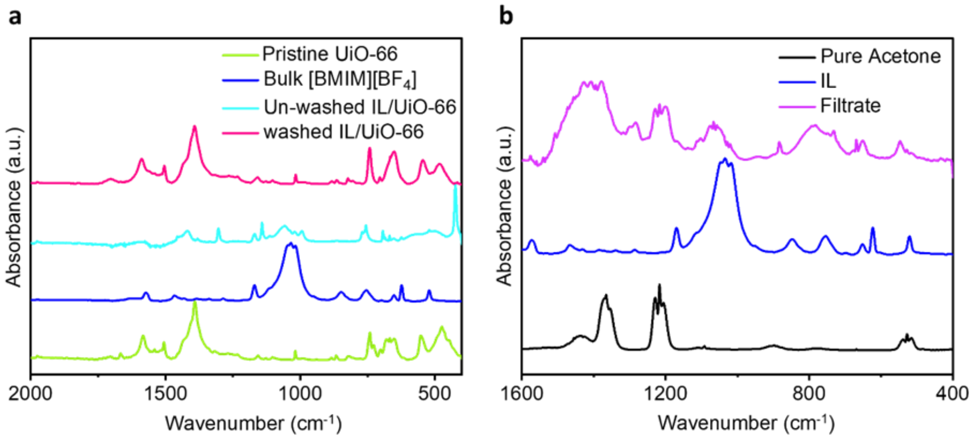

Figure S5. IR spectra of pristine UiO-66, [BMIM][BF$_4$], and [BMIM][BF$_4$]/UiO-66 before and after washing with acetone. **(a)** [BMIM][BF$_4$]/UiO-66 samples before and after washing with acetone solvent, **(b)** filtrate obtained after the washing of [BMIM][BF$_4$]/UiO-66 with acetone.

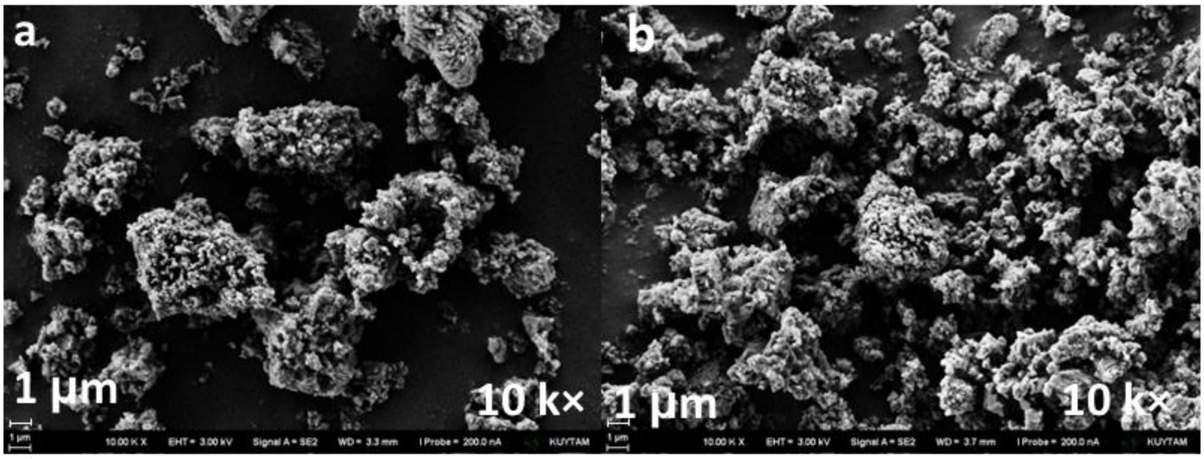

Figure S6. SEM images of **(a)** UiO-66 and **(b)** [BMIM][BF_4]/UiO-66 composite obtained at magnifications of 10 k×.

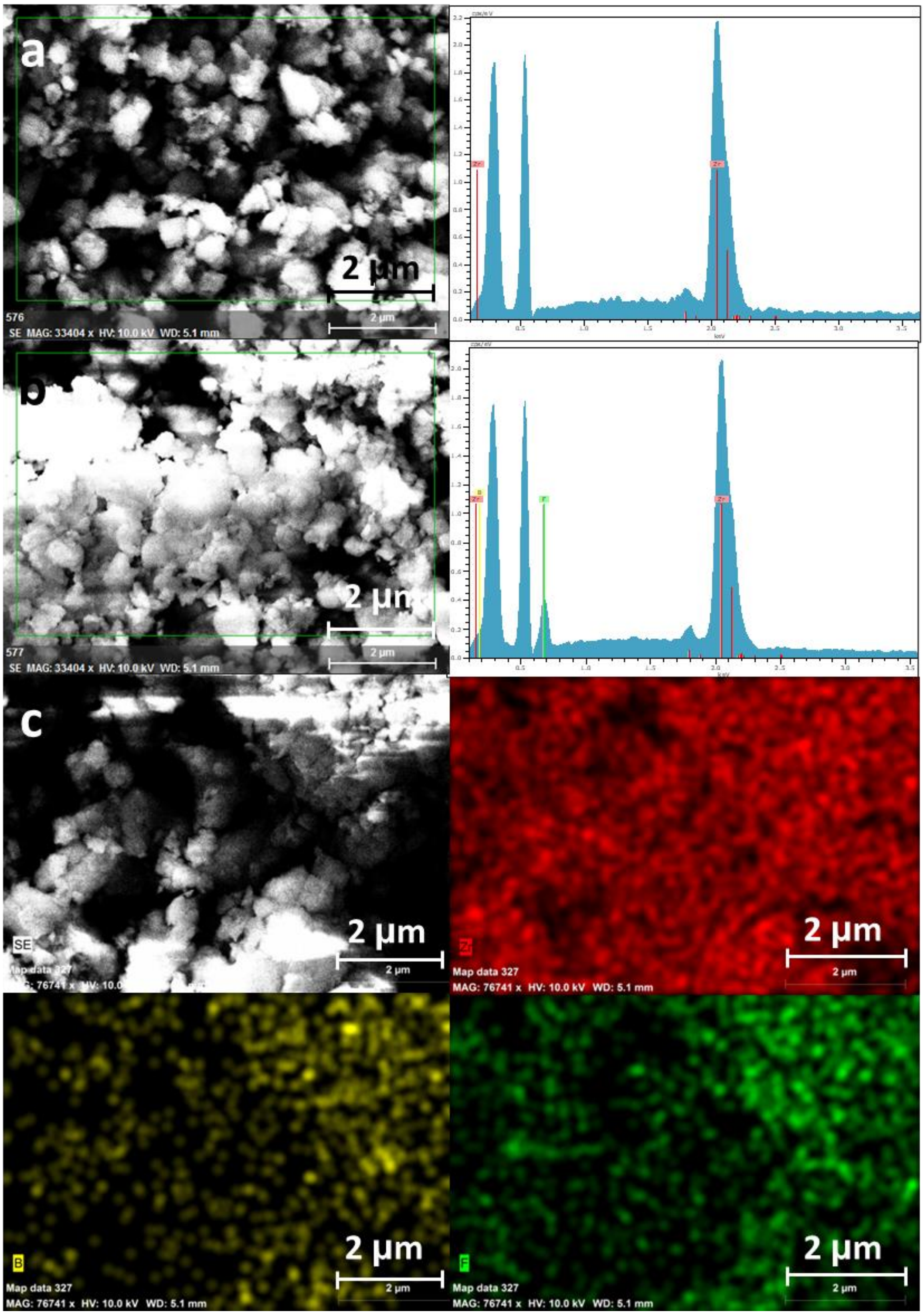

Figure S7. **(a)** SEM image and EDX spectra of pristine UiO-66, **(b)** SEM image and EDX spectra of [BMIM][BF_4]/UiO-66 composite, **(c)** elemental mapping of [BMIM][BF_4]/UiO-66 composite and distribution of zirconium (Zr), boron (B) and fluorine (F) elements on selected area.

To have insights into IL-MOF interactions, an IR spectroscopy-based analysis was performed on the UiO-66, [BMIM][BF_4]/UiO-66 composite, and bulk [BMIM][BF_4], as shown in **Figure 6d** of the main manuscript. Characteristic IR vibrational bands of pristine UiO-66

located at 1584, 1392, 741, and 553 cm^{-1} are consistent with the literature.[1] The bands at 553 and 741 cm^{-1} were associated with the asymmetric stretching vibrations of the Zr–(OC) and Zr–O metal bond, while the sharp peaks located at approximately 1392 and 1584 cm^{-1} were associated with symmetric and asymmetric (O−C−O) stretching vibration of the carboxylate group, respectively.[35, 36] IR spectra of [BMIM][BF_4]/UiO-66 composite illustrated blue-shifts in the vibrational mode of Zr–(OC) and Zr–O, from 553 and 741 cm^{-1} to 543 and 745 cm^{-1}. The blue-shifts demonstrated possible interactions of IL with the metal nodes of MOF. In the case of bulk [BMIM][BF_4], characteristic IR vibrational bands of cation located at 2938, 1467, 1119, and 1017 cm^{-1} were identified as imidazolium ring $\nu(CH_3)_{sym}$, imidazolium ring $\delta(CH_3)$ wagging, $\delta(C_2H)/\delta(C_4H)/\delta(C_5H)$ on the imidazolium ring, butyl $\delta(HC_4H)$ (or $\nu(BF_4)_{asym}$),[14, 37] respectively, while the characteristic IR vibrational bands of the anion located at approximately 1046 and 1033 cm^{-1} were associated with $\nu(BF_4)_{asym}$, and $\nu(BF_4)_{sym}$, respectively. It was observed that the vibrational modes belonging to anion were blue-shifted to higher wavenumbers, from 1033 cm^{-1} to 1039 cm^{-1}, whereas imidazolium cation related characteristic IR band red-shifted, from 1466 cm^{-1} to 1457 cm^{-1}, upon the incorporation of IL into the UiO-66, indicating the strengthening of the B–F bond in $[BF_4]^-$ anion and weakening of the intermolecular bonds in $[BMIM]^+$ cation. Hence, these shifts obtained from the IR spectra of the IL/MOF composite further verify the presence of the intermolecular interactions between IL molecules and MOF cages. Accordingly, IL-MOF molecular interactions can be interpreted as between the $[BMIM]^+$ cation of [BMIM][BF_4] and the Zr metal nodes of the UiO-66.

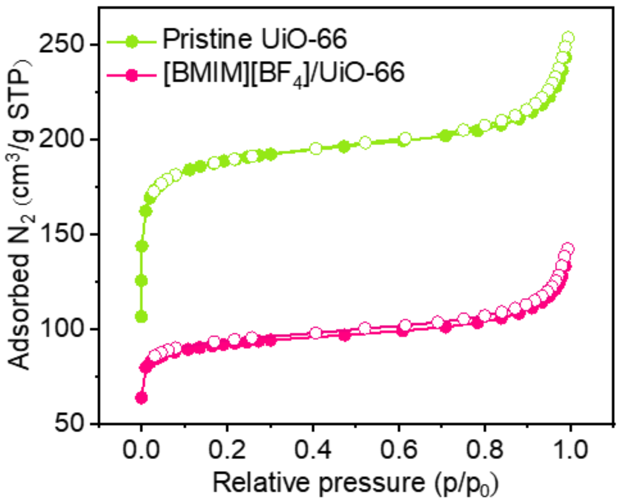

Figure S8. N_2 adsorption-desorption isotherms of pristine UiO-66 and [BMIM][BF_4]/UiO-66 composite obtained at −196 °C. Filled circles represent adsorption, and empty circles represent desorption.

N_2 adsorption-desorption isotherms of pristine UiO-66 and [BMIM][BF_4]/UiO-66 composite given in **Figure S8** were associated with Type I isotherm, and results tabulated in **Table S5** illustrated a decrease in both surface area and pore volume of pristine UiO-66 upon the incorporation of IL. Such a decrease in both surface area and pore volume was expected, considering the pore occupation of MOF by [BMIM][BF_4] molecules resulting in less N_2 uptakes, consistent with the previously reported IL/MOF composite studies.[4, 15] Accordingly, Conductor-like Screening Model for Realistic Solvents (COSMO-RS) calculations were performed to estimate the solubility of N_2 gas in bulk [BMIM][BF_4] at the BET measurement conditions as given in **Figure S9**. Results demonstrated that the N_2 has almost negligible solubility in [BMIM][BF_4] at the BET measurement conditions. Therefore, the BET analysis can be quantitatively misleading in terms of providing reliable data for IL-incorporated composites because of the poor solubility of N_2 gas in bulk IL, however, they indicate the successful incorporation of the IL.

Table S5. BET surface areas and pore volumes of pristine UiO-66 and [BMIM][BF_4]/UiO-66 composite.

Sample	S_{BET} (m^2/g)	V_{pore} (m^3/g)
UiO-66	580	0.1241
[BMIM][BF_4]/UiO-66	284	0.0900

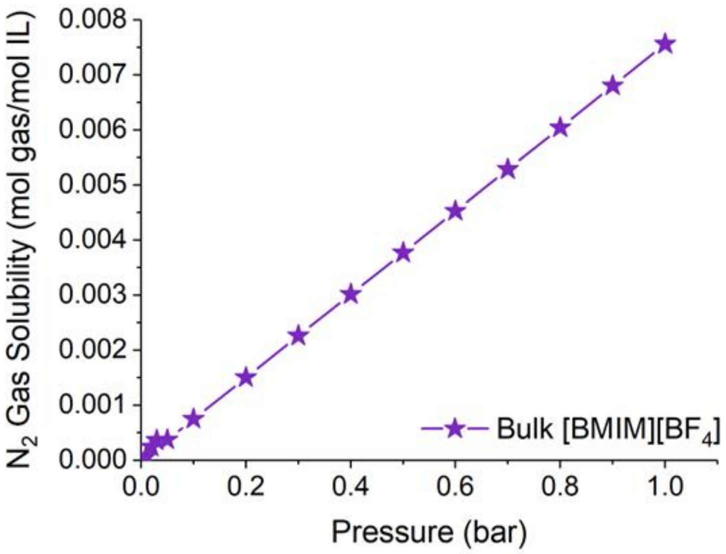

Figure S9. Solubility of N_2 in bulk [BMIM][BF_4] computed by COSMO-RS calculations in the range of 0.001-1 bar at -196 ℃.

High-pressure volumetric gas adsorption measurements. Volumetric gas adsorption measurements of pristine UiO-66 and [BMIM][BF_4]/UiO-66 composite were performed for CO_2 and N_2 using Micromeritics (Particulate Systems) High-Pressure Volumetric Analyzer (HPVA II-200). Approximately 150 mg of sample was used for the measurements. Prior to the measurements, samples were activated overnight for approximately 12 h at 150 °C with a vacuum pressure of 10^{-6} bar. After the activation step, CO_2 and N_2 adsorption isotherms were obtained at 298 K between the pressure range of 0.1-1 bar. Regeneration of the samples were made by degassing at 150 °C for 3 h in between each analysis. Isotherms were fitted to Dual-site Langmuir-Freundlich (DSLF) model and Dual-site Langmuir (DSL) model as given in the following equations: DSLF $\rightarrow n(P)=q_1\times((k_1\times P)^{n_1}/(1+k_1\times P)^{n_1})+q_2\times((k_2\times P)^{n_2}/(1+k_2\times P)^{n_2})$. DSL $\rightarrow n(P)=q_1\times((k_1\times P)/(1+k_1\times P))+q_2\times((k_2\times P)/(1+k_2\times P))$. Here, n(P) represents adsorption quantity under equilibrium temperature, and P represents pressure. q_1 and q_2 represent the maximum adsorption capacity on sites #1 and #2, while k_1 and k_2 are the temperature-dependent equilibrium constants. n_1 and n_2 are the corresponding fitting parameters. The fittings were made by using IAST++ software.[38]

Table S6. Isotherm fitting parameters of pristine UiO-66 and [BMIM][BF_4]/UiO-66 composite.

	**Dual-Site Langmuir-Freundlich Model**							
**Adsorbent**	Gas	q_1 (cc/g)	k_1 (1/mbar)	n_1^*	q_2 (cc/g)	k_2 (1/mbar)	n_2^*	R^2
**Pristine UiO-66**	N_2	5.56	0.56	1.04	6.08	0.38	1.89	0.99
**[BMIM][BF_4]/ UiO-66**	CO_2	5.98	2.65	0.97	46.44	0.42	1.01	0.99
	N_2	1.46	0.97	1.84	1.20	0.90	0.98	1
	**Dual-Site Langmuir Model**							
**Adsorbent**	Gas	q_1 (cc/g)	k_1 (1/mbar)	q_2 (cc/g)	k_2 (1/mbar)	R^2		
**Pristine UiO-66**	CO_2	92.07	0.50	6.06	5.75	0.99		

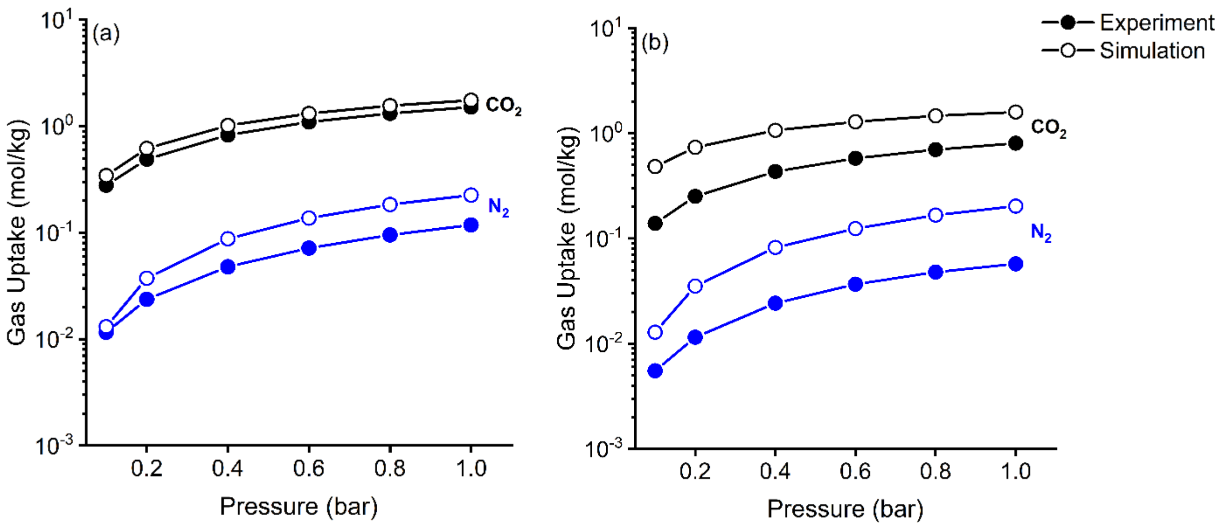

Figure S10. Comparison of experimental (empty circles) and simulation (filled circles) single component adsorption isotherms of CO_2 and N_2 as a function of pressure at 298 K for **(a)** pristine and **(b)** [BMIM][BF$_4$]-incorporated UiO-66.

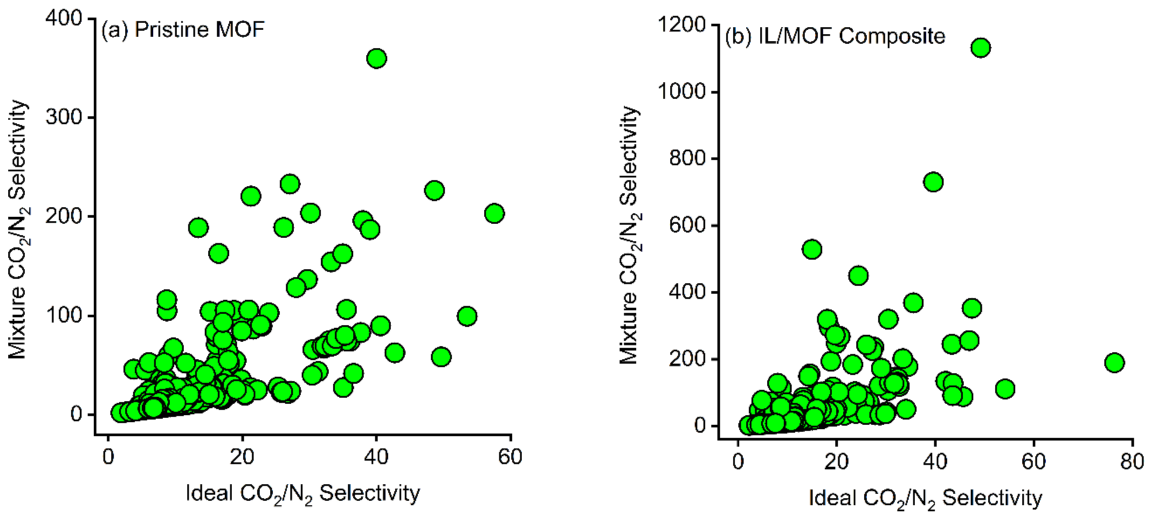

Figure S11. Comparison of ideal and mixture (15/85) CO_2/N_2 selectivities for **(a)** pristine MOFs, **(b)** IL/MOF composites at 1 bar, 298 K.

References

1. Zeeshan, M.; Gulbalkan, H. C.; Durak, O.; Haslak, Z. P.; Unal, U.; Keskin, S.; Uzun, A., An Integrated Computational–Experimental Hierarchical Approach for the Rational Design of an IL/UiO-66 Composite Offering Infinite CO_2 Selectivity. *Adv. Funct. Mater.* **2022**, 2204149.
2. Sezginel, K. B.; Keskin, S.; Uzun, A., Tuning the Gas Separation Performance of CuBTC by Ionic Liquid Incorporation. *Langmuir* **2016,** *32* (4), 1139-1147.
3. Polat, H. M.; Zeeshan, M.; Uzun, A.; Keskin, S., Unlocking CO_2 separation performance of ionic liquid/CuBTC composites: Combining experiments with molecular simulations. *Chem. Eng. J.* **2019,** *373*, 1179-1189.
4. Zeeshan, M.; Gulbalkan, H. C.; Haslak, Z. P.; Keskin, S.; Uzun, A., Doubling CO_2/N_2 Separation Performance of CuBTC by Incorporation of 1-n-ethyl-3-methylimidazolium diethyl Phosphate. *Microporous Mesoporous Mater.* **2021,** *316*, 110947.
5. Oliveira, L. T.; Goncalves, R. V.; Gonçalves, D. V.; de Azevedo, D. C. S.; Pereira de Lucena, S. M., Superior Performance of Mesoporous MOF MIL-100 (Fe) Impregnated with Ionic Liquids for CO_2 Adsorption. *J. Chem. Eng. Data* **2019,** *64* (5), 2221-2228.
6. Kulak, H.; Polat, H. M.; Kavak, S.; Keskin, S.; Uzun, A., Improving CO_2 Separation Performance of MIL-53 (Al) by Incorporating 1-n-Butyl-3-Methylimidazolium Methyl Sulfate. *Energy Technol.* **2019,** *7* (7), 1900157.
7. Kavak, S.; Polat, H. M.; Kulak, H.; Keskin, S.; Uzun, A., MIL-53 (Al) as a Versatile Platform for Ionic-Liquid/MOF Composites to Enhance CO_2 Selectivity over CH_4 and N_2. *Chem. Asian J.* **2019,** *14* (20), 3655-3667.
8. Ma, J.; Ying, Y.; Guo, X.; Huang, H.; Liu, D.; Zhong, C., Fabrication of Mixed-Matrix Membrane Containing Metal–Organic Framework Composite with Task-Specific Ionic Liquid for Efficient CO_2 Separation. *J. Mater. Chem. A* **2016,** *4* (19), 7281-7288.
9. Kinik, F. P.; Altintas, C.; Balci, V.; Koyuturk, B.; Uzun, A.; Keskin, S., [BMIM][PF_6] Incorporation Doubles CO_2 Selectivity of ZIF-8: Elucidation of Interactions and Their Consequences on Performance. *ACS Appl. Mater. Interfaces* **2016,** *8* (45), 30992-31005.
10. Ferreira, T. J.; Ribeiro, R. P.; Mota, J. P.; Rebelo, L. P.; Esperança, J. M.; Esteves, I. A., Ionic Liquid-Impregnated Metal–Organic Frameworks for CO_2/CH_4 Separation. *ACS Appl. Nano Mater.* **2019,** *2* (12), 7933-7950.
11. Zeeshan, M.; Nozari, V.; Yagci, M. B.; Isık, T.; Unal, U.; Ortalan, V.; Keskin, S.; Uzun, A., Core–Shell Type Ionic Liquid/Metal Organic Framework Composite: an Exceptionally High CO_2/CH_4 Selectivity. *J. Am. Chem. Soc.* **2018,** *140* (32), 10113-10116.
12. Ban, Y.; Li, Z.; Li, Y.; Peng, Y.; Jin, H.; Jiao, W.; Guo, A.; Wang, P.; Yang, Q.; Zhong, C., Confinement of Ionic Liquids in Nanocages: Tailoring the Molecular Sieving Properties of ZIF-8 for Membrane-Based CO_2 Capture. *Angew. Chem. Int. Ed.* **2015,** *54* (51), 15483-15487.
13. Ferreira, T. J.; Vera, A. T.; De Moura, B. A.; Esteves, L. M.; Tariq, M.; Esperança, J. M.; Esteves, I. A., Paramagnetic Ionic Liquid/Metal Organic Framework Composites for CO_2/CH_4 and CO_2/N_2 Separations. *Front. Chem.* **2020,** *8*, 590191.
14. Koyuturk, B.; Altintas, C.; Kinik, F. P.; Keskin, S.; Uzun, A., Improving Gas Separation Performance of ZIF-8 by [BMIM][BF_4] Incorporation: Interactions and Their Consequences on Performance. *J. Phys. Chem. C* **2017,** *121* (19), 10370-10381.
15. Zeeshan, M.; Kulak, H.; Kavak, S.; Polat, H. M.; Durak, O.; Keskin, S.; Uzun, A., Influence of Anion Size and Electronic Structure on the Gas Separation Performance of Ionic Liquid/ZIF-8 Composites. *Microporous Mesoporous Mater.* **2020,** *306*, 110446.
16. Zeeshan, M.; Keskin, S.; Uzun, A., Enhancing CO_2/CH_4 and CO_2/N_2 Separation Performances of ZIF-8 by Post-Synthesis Modification with [BMIM][SCN]. *Polyhedron* **2018,** *155*, 485-492.
17. Hussain, S.; Dong, H.; Zhang, Y.; Zhan, G.; Zeng, S.; Duan, H.; Zhang, X., Impregnation of 1-n-Butyl-3-methylimidazolium Dicyanide [BMIM][DCA] into ZIF-8 as a Versatile Sorbent for Efficient and Selective Separation of CO_2. *Ind. Eng. Chem. Res.* **2021,** *61* (1), 706-715.
18. Mohamedali, M.; Ibrahim, H.; Henni, A., Incorporation of Acetate-Based Ionic Liquids into a Zeolitic Imidazolate Framework (ZIF-8) as Efficient Sorbents for Carbon Dioxide Capture. *J. Chem. Eng.* **2018,** *334*, 817-828.

19. Philip, F. A.; Henni, A., Enhancement of Post-Combustion CO_2 Capture Capacity by Incorporation of Task-Specific Ionic Liquid into ZIF-8. *Microporous and Mesoporous Materials* **2022,** *330*, 111580.
20. Moghadam, P. Z.; Li, A.; Wiggin, S. B.; Tao, A.; Maloney, A. G.; Wood, P. A.; Ward, S. C.; Fairen-Jimenez, D., Development of a Cambridge Structural Database Subset: a Collection of Metal–Organic Frameworks for Past, Present, and Future. *Chem. Mater.* **2017,** *29* (7), 2618-2625.
21. Polat, H. M.; Kavak, S.; Kulak, H.; Uzun, A.; Keskin, S., CO_2 Separation from Flue Gas Mixture Using [BMIM][BF_4]/MOF Composites: Linking High-Throughput Computational Screening with Experiments. *Chem. Eng. J.* **2020,** *394*, 124916.
22. Rappe, A. K.; Goddard, W. A., Charge Equilibration for Molecular Dynamics Simulations. *J. Phys. Chem.* **1991,** *95* (8), 3358-3363.
23. Tee, L. S.; Gotoh, S.; Stewart, W. E., Molecular Parameters for Normal Fluids. Lennard-Jones 12-6 Potential. *Ind. Eng. Chem.* **1966,** *5* (3), 356-363.
24. Ewald, P. P., Die Berechnung Optischer und Elektrostatischer Gitterpotentiale. *Annalen der physik* **1921,** *369* (3), 253-287.
25. Mayo, S. L.; Olafson, B. D.; Goddard, W. A., DREIDING: A Generic Force Field for Molecular Simulations. *J. Phys. Chem.* **1990,** *94* (26), 8897-8909.
26. Potoff, J. J.; Siepmann, J. I., Vapor–iquid Equilibria of Mixtures Containing Alkanes, Carbon Dioxide, and Nitrogen. *AIChE J.* **2001,** *47* (7), 1676-1682.
27. Makrodimitris, K.; Papadopoulos, G. K.; Theodorou, D. N., Prediction of Permeation Properties of CO_2 and N_2 through Silicalite via Molecular Simulations. *J. Phys. Chem. B* **2001,** *105* (4), 777-788.
28. Willems, T. F.; Rycroft, C. H.; Kazi, M.; Meza, J. C.; Haranczyk, M., Algorithms and Tools for High-Throughput Geometry-Based Analysis of Crystalline Porous Materials. *Microporous Mesoporous Mater.* **2012,** *149* (1), 134-141.
29. Daglar, H.; Keskin, S., Combining Machine Learning and Molecular Simulations to Unlock Gas Separation Potentials of MOF Membranes and MOF/Polymer MMMs. *ACS Appl. Mater. Interfaces* **2022**.
30. Yang, P.; Zhang, H.; Lai, X.; Wang, K.; Yang, Q.; Yu, D., Accelerating the Selection of Covalent Organic Frameworks with Automated Machine Learning. *ACS omega* **2021,** *6* (27), 17149-17161.
31. Pedregosa, F.; Varoquaux, G.; Gramfort, A.; Michel, V.; Thirion, B.; Grisel, O.; Blondel, M.; Prettenhofer, P.; Weiss, R.; Dubourg, V., Scikit-learn: Machine Learning in Python. *J Mach Learn Res* **2011,** *12*, 2825-2830.
32. Momma, K.; Izumi, F., VESTA 3 for Three-Dimensional Visualization of Crystal, Volumetric and Morphology Data. *J. Appl. Crystallogr.* **2011,** *44* (6), 1272-1276.
33. Wojdyr, M., Fityk: A General-Purpose Peak Fitting Program. *J. Appl. Crystallogr.* **2010,** *43* (5-1), 1126-1128.
34. Iacomi, P.; Lee, U.-H.; Valekar, A. H.; Chang, J.-S.; Llewellyn, P. L., Investigating the Effect of Alumina Shaping on the Sorption Properties of Promising Metal–Organic Frameworks. *RSC Adv.* **2019,** *9* (13), 7128-7135.
35. Durak, Ö.; Kulak, H.; Kavak, S.; Polat, H. M.; Keskin, S.; Uzun, A., Towards Complete Elucidation of Structural Factors Controlling Thermal Stability of IL/MOF Composites: Effects of Ligand Functionalization on MOFs. *J. Phys. Condens. Matter* **2020,** *32* (48), 484001.
36. Jin, Z.; Yang, H., Exploration of Zr–Metal–Organic Framework as Efficient Photocatalyst for Hydrogen Production. *Nanoscale Res. Lett.* **2017,** *12* (1), 1-10.
37. Babucci, M.; Balci, V.; Akçay, A.; Uzun, A., Interactions of [BMIM][BF_4] with Metal Oxides and Their Consequences on Stability Limits. *J. Phys. Chem. C* **2016,** *120* (36), 20089-20102.
38. Lee, S.; Lee, J. H.; Kim, J., User-Friendly Graphical User Interface Software for Ideal Adsorbed Solution Theory Calculations. *Korean J Chem Eng* **2018,** *35* (1), 214-221.
